# Gas Chromatography–Mass Spectrometry Analysis of Volatile Organic Compounds from Three Endemic *Iris* Taxa: Headspace Solid-Phase Microextraction vs. Hydrodistillation

**DOI:** 10.3390/molecules29174107

**Published:** 2024-08-29

**Authors:** Maja Friščić, Željan Maleš, Ivanka Maleš, Ivan Duka, Ani Radonić, Božena Mitić, Dario Hruševar, Sandra Jurić, Igor Jerković

**Affiliations:** 1 Department of Pharmaceutical Botany, University of Zagreb Faculty of Pharmacy and Biochemistry, Ante Kovačića 1, 10 000 Zagreb, Croatia; maja.friscic@pharma.unizg.hr (M.F.); zeljan.males@pharma.unizg.hr (Ž.M.); iv.duka@gmail.com (I.D.); 2 Department of Pharmacy, University of Split School of Medicine, Šoltanska 2A, 21 000 Split, Croatia; ivanka.males@mefst.hr; 3 Medical School Karlovac, Dr Andrije Štampara 5, 47 000 Karlovac, Croatia; 4 Department of Organic Chemistry, University of Split Faculty of Chemistry and Technology, Ruđera Boškovića 35, 21 000 Split, Croatia; radonic@ktf-split.hr; 5 Division of Botany, Department of Biology, University of Zagreb Faculty of Science, Marulićev trg 9a, 10 000 Zagreb, Croatia; bozena.mitic@biol.pmf.hr (B.M.); dario.hrusevar@biol.pmf.hr (D.H.); 6 Department of Organic Chemistry, University of Zagreb Faculty of Pharmacy and Biochemistry, Ante Kovačića 1, 10 000 Zagreb, Croatia; sandra.juric@pharma.unizg.hr

**Keywords:** *Iris adriatica*, *Iris illyrica*, *Iris pseudopallida*, essential oil, scent components, fragrance components, irones, orris root, orris butter, myristic acid

## Abstract

*Iris* taxa are sources of valuable essential oils obtained from aged rhizomes used by various industries, including pharmacy, cosmetic, perfume, and food industry, in which irones are the most important aroma components. In this study, volatile organic compounds (VOCs) obtained from dried rhizomes of three endemics from Croatia, *Iris pseudopallida*, *I. illyrica*, and *I. adriatica*, were studied. The VOCs were isolated by three different methods: headspace solid-phase microextraction (HS–SPME) using divinylbenzene/carboxene/polydimethylsiloxane (DVB/CAR/PDMS) fiber or polydimethylsiloxane/divinylbenzene (PDMS/DVB) fiber, and hydrodistillation (HD). The samples were analyzed by gas chromatography–mass spectrometry (GC–MS). In five out of six samples, the main compounds detected by HS–SPME were perilla aldehyde, butan-2,3-diol, acetic acid, 2-phenylethanol, benzyl alcohol, hexanal, and nonanal, while 6-methylhept-5-en-2-one, *trans*-caryophyllene, and ethanol were common for all studied samples. The former VOCs were absent from the oldest, irone-rich *I. pseudopallida* sample, mainly characterized by *cis*-α-irone (43.74–45.76%). When using HD, its content was reduced (24.70%), while docosane prevailed (45.79%). HD yielded predominantly fatty acids, including myristic, common for all studied taxa (4.20–97.01%), and linoleic (40.69%) and palmitic (35.48%) as the major VOCs of *I. adriatica* EO. The performed GC–MS analyses of EOs, in combination with HS–SPME/GC–MS, proved to be useful for gaining a better insight into *Iris* VOCs.

## 1. Introduction

In recent years, there has been increased interest in medicinal and aromatic plants since they are sources of many bioactive compounds with diverse pharmacological activities and medicinal applications [1]. Particularly interesting for pharmacy and the cosmetic industry, due to their diverse therapeutic and cosmeceutical properties, are essential oils (EOs), mixtures of volatile organic compounds (VOCs) that are obtained by different distillation methods from plant-based raw materials [2]. Dried rhizomes of various *Iris* L. species (mainly *Iris pallida* Lam., *I. × germanica* L., and *I. florentina* L.), also known as orris roots, are resources of a semi-solid essential oil (EO), i.e., “orris oil” or “orris butter”, which is especially valued in perfumery but is also used by the food industry to flavor soft and alcoholic beverages (e.g., rum, gin, and vermouth) and sweets [3,4]. This is not only because of the pleasant violet-like smell coming from its volatile constituents [3] but also because of their powerful fixative properties [4]. The name “orris butter” comes from the fact that the EO has a high share of myristic (tetradecanoic, C_14_H_28_O_2_) acid (around 65%) and other saturated fatty acids, such as lauric (dodecanoic, C_12_H_24_O_2_) acid and palmitic (hexadecanoic, C_16_H_32_O_2_) acid [3], the melting points of which are above the human body temperature [5]. However, the characteristic smell of the oil is mainly not due to the presence of fatty acids but to the presence of irones, C_14_ monocyclic ketones, which are believed to be formed by oxidative degradation from their triterpenic precursors iridals during rhizome maturation (aging) and are not present in fresh plant material. A storage period of about 2-to-5 years is believed to be required for rhizomes of irises to achieve their maximum irone content and fullness of the scent, resulting in high-quality EO [3,4].

The determined amounts (yields) and chemical composition of VOCs may vary depending on the used isolation technique. Hydrodistillation (HD) is one of the most frequently used techniques for the extraction of VOCs from plant material [6], which is environmentally friendly [7]. On the other hand, headspace solid-phase microextraction (HS–SPME) represents a relatively novel, easy, effective, and convenient technique, which allows the extraction of VOCs on the fiber coating depending on their concentrations in the sample headspace and can be used for rapid isolation of VOCs from plant material [8,9]. HS–SPME has been extensively used for the extraction of VOCs, without the need for the sample pre-treatment [9,10].

The genus *Iris* (Iridaceae) has about 300 species distributed worldwide, with the majority of endemic species located in Asia and the Mediterranean area [11]. Many of these species are used in traditional medicine, e.g., *I. germanica* to reduce the activity of smooth muscles, *I. dichotoma* Pall. to treat respiratory and rheumatic disorders, and *I. florentina* for digestive and metabolic disorders [12,13,14]. Moreover, dried rhizomes of various *Iris* species were utilized collectively in order to create toothpowders and ease children’s teething pain [15]. Due to the presence of different bioactive compounds, such as flavones, isoflavones, benzophenones, and xanthones, *Iris* extracts have been reported to have many biological activities [12,14,15,16]. These include antioxidative, anticancer, antibacterial, anti-inflammatory, antifungal, antiviral, anthelmintic, antidiabetic, neuroprotective, hepatoprotective, hypolipidemic, and other activities [14,15,16,17,18,19]. Nevertheless, species from the genus *Iris* are rich sources of EOs, which can be found in a variety of plant parts, mostly in the rhizomes, but also in the roots, seeds, leaves and flowers, and may be used in aromatherapy for their sedative properties [14,20].

Reports on the composition of EOs obtained by HD have been published for various *Iris* species, including *I. persica* L. [21], *I. germanica*, *I. aurantiaca* Dinsm., *I. barnumae* Bak, *I. bostrensis* Mouterde [22], and *I. bulleyana* Dykes [23]. Moreover, the compositions of VOCs detected by HS–SPME have been reported for *I. germanica* [24,25], *I. pallida* and *I. pumila* L. [25], and *I. lactea* var. *chinensis* (Fisch.) Koidz in three flowering phases [26]. However, neither the composition of headspace VOCs extracted by HS–SPME nor of EOs obtained by HD of *Iris pseudopallida* Trinajstić, *I. illyrica* Tomm., and *I. adriatica* Trinajstić ex Mitić have been reported so far.

**Figure 1:**
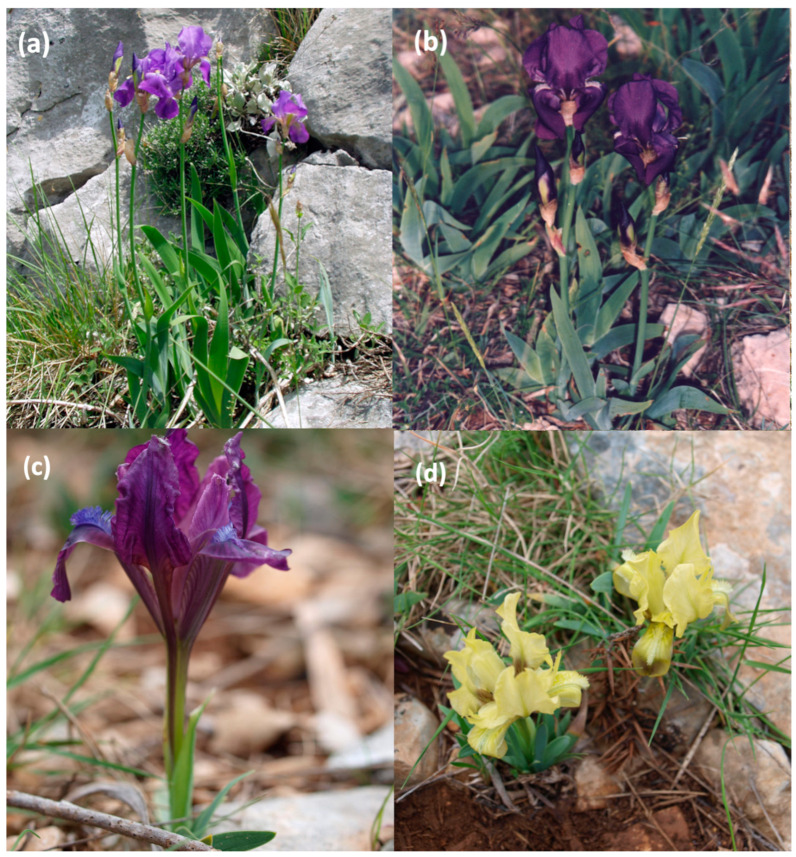
Investigated endemic *Iris* taxa from Croatia: (**a**) *Iris pseudopallida*, (**b**) *I. illyrica*, (**c**) *I. adriatica* (a purple-flowered individual), and (**d**) *I. adriatica* (yellow-flowered individuals). Photo: B. & M. Mitić.

*Iris pseudopallida*, *I. illyrica*. and *I. adriatica* are rare taxa of herbaceous wild perennials native to Croatia and some neighboring areas [16,27,28]. The investigated taxa belong to the so-called “bearded irises” (“Pogoniris”), whereby *I. adriatica* is a dwarf species with single yellow or violet flowers, while the other two taxa are tall with several pale or darker blue or violet flowers (Figure 1). *I. adriatica* is strictly endemic (widespread on the Croatian coast, in the hinterland and on the islands of Central Dalmatia), while *I. illyrica* (widespread on the mountainous coasts and hinterland and islands of the Northern Adriatic, from Italy and Northern Slovenia and Croatia to the Southern Velebit in Croatia and the Eastern Dinaric area of Bosnia and Hercegovina) and *I. pseudopallida* (widespread on the coasts, valleys and on the islands of the Southern Adriatic, from the Kozjak mountain in Croatia to the south of Croatia, Montenegro, and Albania) are subendemic taxa [27].

The current study aimed to identify, for the first time, the characteristic VOCs of *Iris pseudopallida*, *I. illyrica*, and *I. adriatica* extracted by HS–SPME and HD. The samples were collected from various locations in Croatia, and their VOCs compositions were analyzed using gas chromatography and mass spectrometry (GC–MS). Reported results on the EO compositions or VOC profiles of *Iris* species are usually based on analyses of single populations [21,22,23,24,26,29]. Therefore, the novelty of this study is also related to a larger number of samples of investigated *Iris* taxa (three for *I*. *pseudopallida* and two for *I. illyrica*), taking into account possible differences in environmental and geographic conditions, which may affect the production of VOCs [30].

## 2. Results

### 2.1. HS–SPME/GC–MS Analysis

The chemical composition of the extracted analyte is influenced by the compound volatility, the polarity, and the characteristics of the fiber coating [31]. Consequently, in the present study, two fibers, divinylbenzene/carboxene/polydimethylsiloxane (DVB/CAR/PDMS) and polydimethylsiloxane/divinylbenzene (PDMS/DVB), were selected for HS–SPME. These fiber choices were based on their suitability for untargeted analysis, as was previously reported [32].

#### 2.1.1. PDMS/DVB Fiber

Headspace VOCs of three populations of *I. pseudopallida*, two populations of *I. illyrica* and one of *I. adriatica*, were isolated and analyzed by HS–SPME/GC–MS, using PDMS/DVB fiber. A total of 77 compounds were identified, accounting for 84.00–96.22% of the total VOC content (Table 1). Oxygenated monoterpenes were the most dominant compounds in *I. pseudopallida* from Bast (*I. pseudopallida* B, 38.00%) and Topići (*I. pseudopallida* T, 34.40%), as well as in *I. illyrica* from Vir (*I. illyrica* V, 23.07%) and Zaton (*I. illyrica* Z, 36.26%) and in *I. adriatica* (43.24%), while norisoprenoids dominated in *I. pseudopallida* from Dubrovnik (*I. pseudopallida* D, 52.93%). The major identified compounds in *I. pseudopallida* B were perilla aldehyde (20.55%), myrtenol (6.33%), α-copaene (5.14%), 2-phenylethanol (4.67%), α-pinene (4.48%), and *trans*-caryophyllene (3.40%). The major identified compounds in *I. pseudopallida* D included *cis*-α-irone (45.76%), *trans*-caryophyllene (7.24%), *cis*-γ-irone (7.17%), nonan-2-one (4.99%), 6-methylhept-5-en-2-one (3.59%), and pentanal (3.28%). Furthermore, the principal identified compounds in *I. pseudopallida* T were perilla aldehyde (17.76%), ethanol (6.86%), butan-2,3-diol (6.38%), acetic acid (6.29%), benzyl alcohol (5.46%), and neryl formate (4.02%). The major identified compounds in *I. illyrica* V were perilla aldehyde (19.72%), acetovanillone (7.12%), acetic acid (6.95%), butan-2,3-diol (5.29%), ethanol (4.79%), and α-copaene (3.62%). The major identified compounds in *I. illyrica* Z were perilla aldehyde (30.00%), butan-2,3-diol (10.27%), 6-methylhept-5-en-2-one (9.47%), 2-phenylethanol (4.76%), ethanol (3.65%), and dihydromyrcenol (3.46%). The primary identified compounds in *I. adriatica* were perilla aldehyde (26.83%), limonene (4.79%), *trans*-caryophyllene (4.51%), 1,8-cineole (3.82%), acetic acid (3.74%), and camphor (3.25%) (Table 1). 

#### 2.1.2. DVB/CAR/PDMS Fiber

Headspace VOCs of three populations of *I. pseudopallida*, two populations of *I. illyrica*, and one of *I. adriatica* were isolated and analyzed by HS–SPME/GC–MS, using DVB/CAR/PDMS fiber. A total of 71 compounds were identified, accounting for 85.67–94.90% of the total VOC content (Table 2). Oxygenated monoterpenes were the main compounds found by PDMS/DVB fiber in *I*. *pseudopallida* B (30.62%), and norisoprenoids in *I*. *pseudopallida* D (51.61%). In *I*. *pseudopallida* T, *I*. *illyrica* V, and *I*. *illyrica* Z, fatty acids, accompanied by fatty acid esters, represented the major group of compounds (35.33%, 30.84%, and 31.02%), while *I. adriatica* VOCs were again characterized by oxygenated monoterpenes (34.32%). The major identified compounds in *I. pseudopallida* B were perilla aldehyde (15.63%), acetic acid (14.29%), myrtenol (6.04%), α-copaene (5.06%), ethanol (4.75%), and 2-phenylethanol (4.31%). The major identified compounds in *I. pseudopallida* D included *cis*-α-irone (43.74%), 6-methylhept-5-en-2-one (9.10%), *cis*-γ-irone (7.87%), *trans*-caryophyllene (5.50%), nonan-2-one (5.11%), and ethanol (3.22%). The principal identified compounds in *I. pseudopallida* T were acetic acid (29.76%), perilla aldehyde (8.09%), butan-2,3-diol (7.05%), benzyl alcohol (5.08%), ethanol (4.70%), and nonanal (2.71%). The major identified compounds in *I. illyrica* V were acetic acid (29.20%), butan-2,3-diol (7.83%), perilla aldehyde (6.47%), ethanol (5.23%), hexanal (4.67%), and acetovanillone (3.85%). The major identified compounds in *I. illyrica* Z were acetic acid (31.02%), perilla aldehyde (17.59%), butan-2,3-diol (11.11%), 6-methylhept-5-en-2-one (5.83%), acetoin (5.27%), and ethanol (4.34%). The primary identified compounds in *I. adriatica* were perilla aldehyde (20.08%), acetic acid (12.00%), furfural (7.91%), ethanol (7.01%), limonene (3.52%), and (furan-2-yl)methanol (3.08%).

### 2.2. HD Analysis

The EOs of *Iris pseudopallida*, *I. illyrica*, and *I. adriatica* were isolated by HD, resulting in the identification of 73 compounds, accounting for 89.31–99.50% of the total VOC content (Table 3). Fatty acids, accompanied by fatty acid esters, were the most abundant EO constituents for *I. pseudopallida* B (37.11%), *I. pseudopallida* T (99.20%), *I. illyrica* Z (81.82%), and *I. adriatica* (85.74%). In *I. pseudopallida* D, alkanes (48.56%) and norisoprenoids (29.44%) were the most abundant groups of compounds. The group of alkanes (55.74%), followed by fatty acids (38.67%), was also characteristic for *I. illyrica* V. The most common individual compound observed in the EO of *I. pseudopallida* B, *I. pseudopallida* T, and *I. illyrica* Z was tetradecanoic acid (31.92–97.01%), whereas (*Z*,*Z*)-octadeca-9,12-dienoic acid (40.69%) and hexadecanoic acid (35.48%) were the major compounds found solely in *I. adriatica* EO. Additionally, dodecanoic acid (1.18–3.90%) and decanoic acid (0.34–2.82%) were detected in all studied samples except for *I. pseudopallida* D. Furthermore, docosane dominated in *I. pseudopallida* D and *I. illyrica* V EO (45.79% and 55.45%). In *I. pseudopallida* D EO, *cis*-α-irone was also found in a high percentage (24.70%), while other constituents occurred in percentages less than 10% in all investigated samples. Among them, several compounds were characteristic of *I. pseudopallida* B EO, including myrtenol (9.60%), terpinen-4-ol (7.27%), α-pinene (5.98%), α-terpineol (3.38%), and 2-phenylethanol (2.20%).

**Figure 2:**
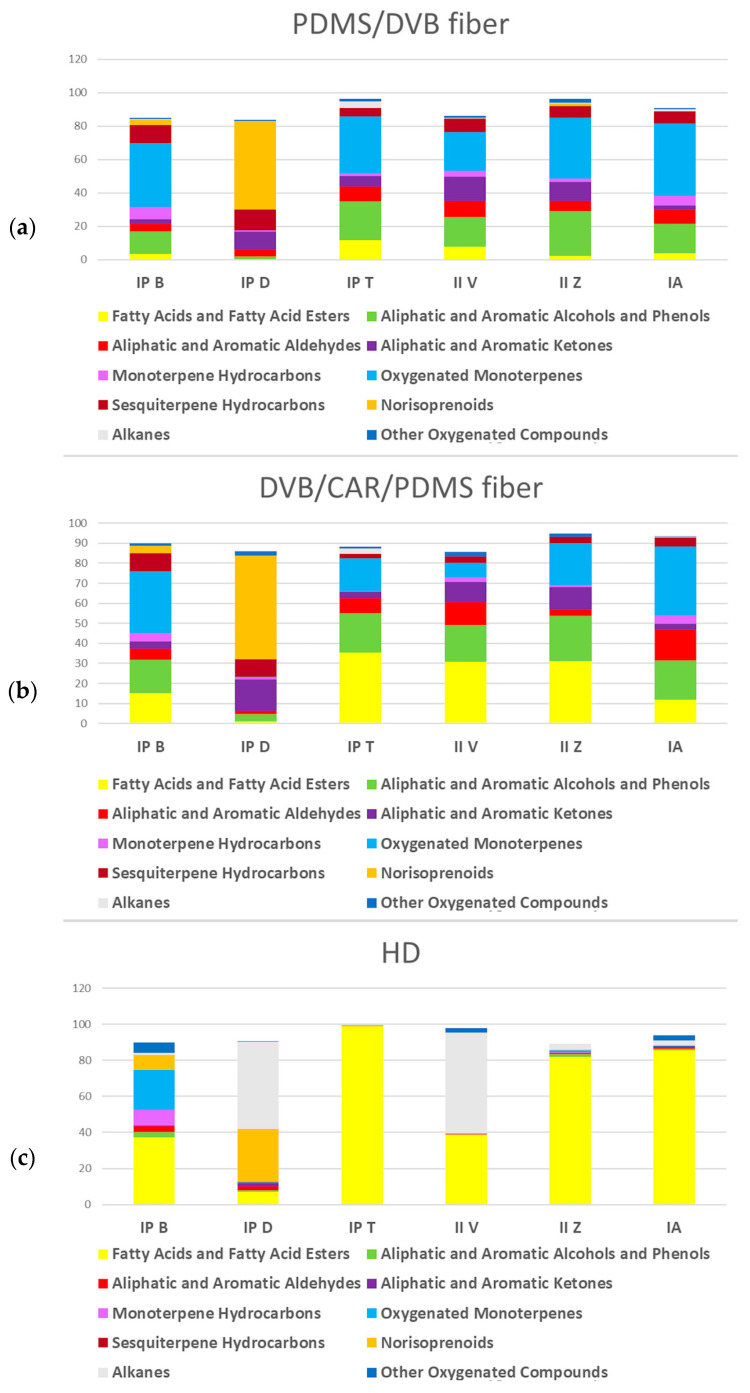
Average percentages of different groups of volatile organic compounds (VOCs) of the six investigated *Iris* samples, obtained by three different methods: (**a**) headspace solid-phase microextraction (HS–SPME) using polydimethylsiloxane/divinylbenzene (PDMS/DVB) fiber, (**b**) HS–SPME using divinylbenzene/carboxene/polydimethylsiloxane (DVB/CAR/PDMS) fiber, and (**c**) hydrodistillation (HD). IP B—*I. pseudopallida* B; IP D—*I. pseudopallida* D; IP T—*I. pseudopallida* T; II V—*I. illyrica* V; II Z—*I. illyrica* Z; IA—*I. adriatica*.

Overall, HD isolated more fatty acids and their esters from *Iris* rhizomes in comparison with HS–SPME, which was expected due to their lower volatility. The same method was more efficient in extraction of alkanes as well. Conversely, HS–SPME using the two different fibers was better for extracting norisoprenoids; aliphatic and aromatic compounds, including alcohols, aldehydes, and ketones; oxygenated monoterpenes; and sesquiterpene hydrocarbons (due to their higher volatility and headspace concentration). Compared with DVB/CAR/PDMS fiber, PDMS/DVB fiber extracted more monoterpene hydrocarbons, oxygenated monoterpenes, and sesquiterpene hydrocarbons, while fatty acids and fatty acid esters were more abundant when using the second fiber (Figure 2). Total ion chromatograms (TICs) of *I. pseudopallida* B are presented in Supplementary Figure S1 as the sample chromatograms.

### 2.3. PCA Analysis of Major VOCs and EOs Constituents

**Figure 3:**
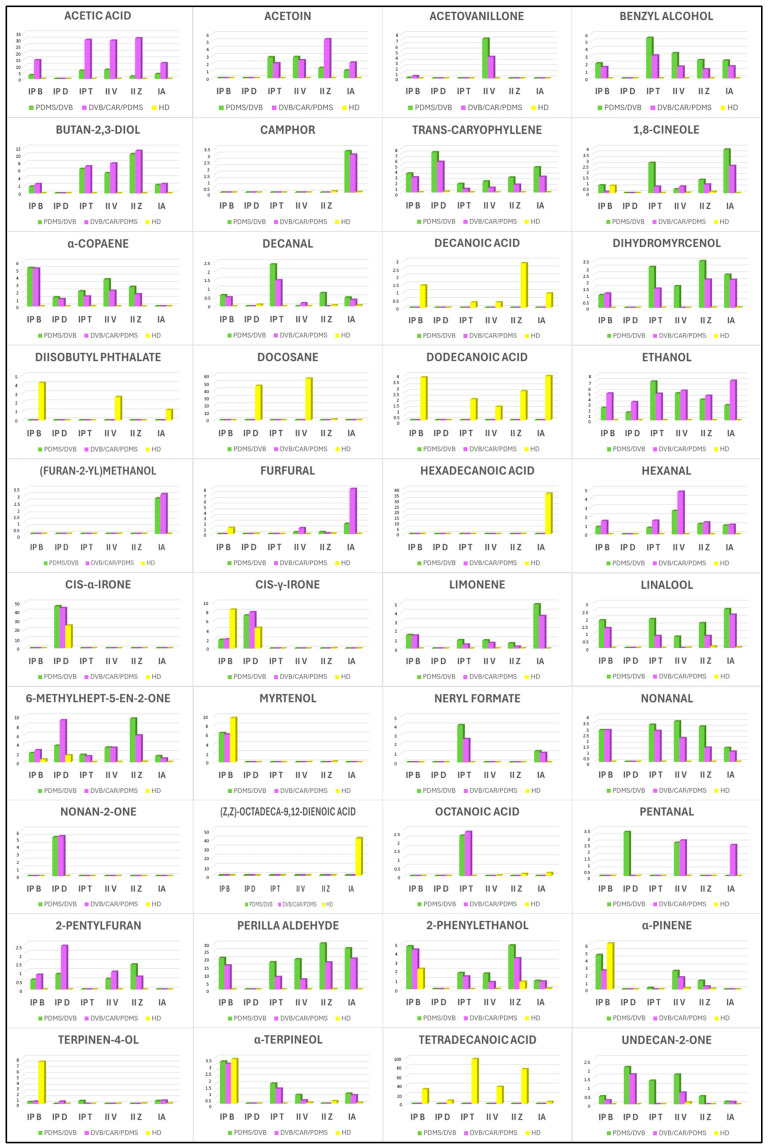
Comparison of the contents of major compounds (content ≥2.0% in at least one sample) of the six investigated *Iris* samples, obtained by three different methods: headspace solid-phase microextraction (HS–SPME) using polydimethylsiloxane/divinylbenzene (PDMS/DVB) fiber or divinylbenzene/carboxene/polydimethylsiloxane (DVB/CAR/PDMS) fiber, or hydrodistillation (HD). IP B—*I. pseudopallida* B; IP D—*I. pseudopallida* D; IP T—*I. pseudopallida* T; II V—*I. illyrica* V; II Z—*I. illyrica* Z; IA—*I. adriatica*.

In order to more easily identify the differences between the VOC compositions and EO compositions of the investigated samples of *I. pseudopallida*, *I. illyrica*, and *I. adriatica* and the interrelationships of the studied taxa, three PCA analyses of their major identified components were performed, one for each method of the sample preparation. In total, 40 major compounds (compounds with a content of 2.0% or more) were identified (Supplementary Table S1; Figure 3).

The VOCs that were more abundant in the samples obtained by extraction with PDMS/DVB fiber included (in alphabetical order): acetovanillone, benzyl alcohol, camphor, *trans*-caryophyllene, 1,8-cineole, α-copaene, decanal, dihydromyrcenol, *cis*-α-irone, limonene, linalool, neryl formate, nonanal, perilla aldehyde, 2-phenylethanol, and undecan-2-one (Figure 3).

More abundant VOCs in the samples obtained by extraction with DVB/CAR/PDMS fiber included acetic acid, butan-2,3-diol, (furan-2-yl)methanol, furfural, hexanal, nonan-2-one, and octanoic acid. Also, in most cases, DVB/CAR/PDMS fiber seemed to be superior to PDMS/DVB fiber when comparing the contents of acetoin, ethanol, pentanal, and 2-pentylfuran (Figure 3).

The compounds that were found exclusively in the samples obtained by HD (essential oils) were the following (in alphabetical order): decanoic acid, diisobutyl phthalate, docosane, dodecanoic acid, hexadecanoic acid, (*Z*,*Z*)-octadeca-9,12-dienoic acid, α-pinene, terpinen-4-ol, and tetradecanoic acid. Moreover, for investigated *Iris* EOs, higher contents of myrtenol and α-terpineol were also observed in comparison to the samples prepared by HS–SPME. Conversely, the presence of acetic acid, acetoin, butan-2,3-diol, ethanol, (furan-2-yl)methanol, hexanal, neryl formate, nonanal, nonan-2-one, pentanal, and 2-pentylfuran was not recorded in any of the samples obtained by HD (Figure 3).

**Figure 4:**
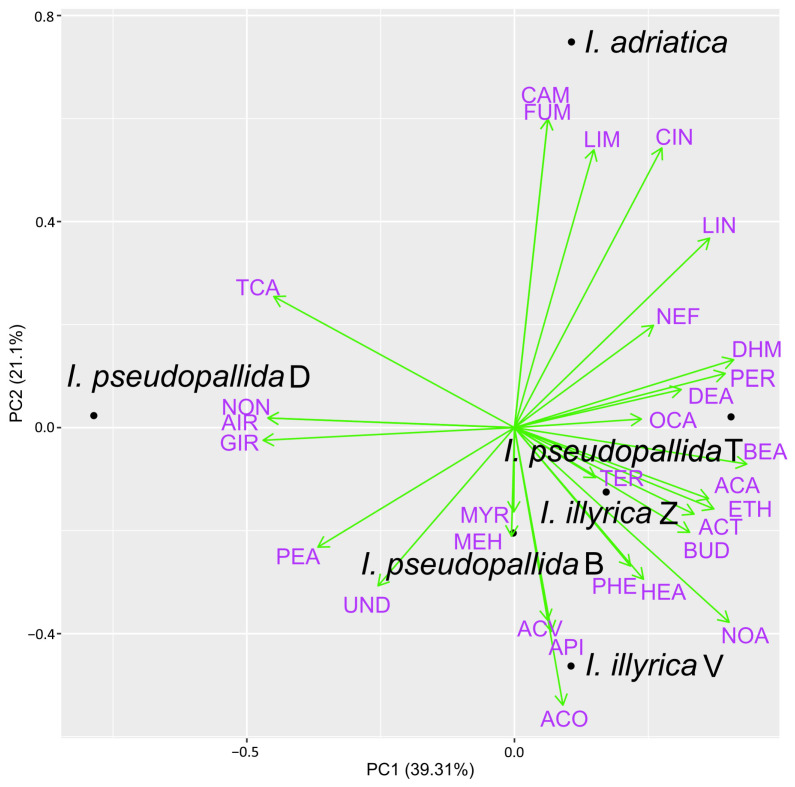
Biplot obtained by principal component analysis of the VOC composition of the six investigated *Iris* samples, based on their major components (average content ≥2.0% in at least one sample), detected using PDMS/DVB fiber; for compound abbreviation, cf. Supplementary Table S1.

**Figure 5:**
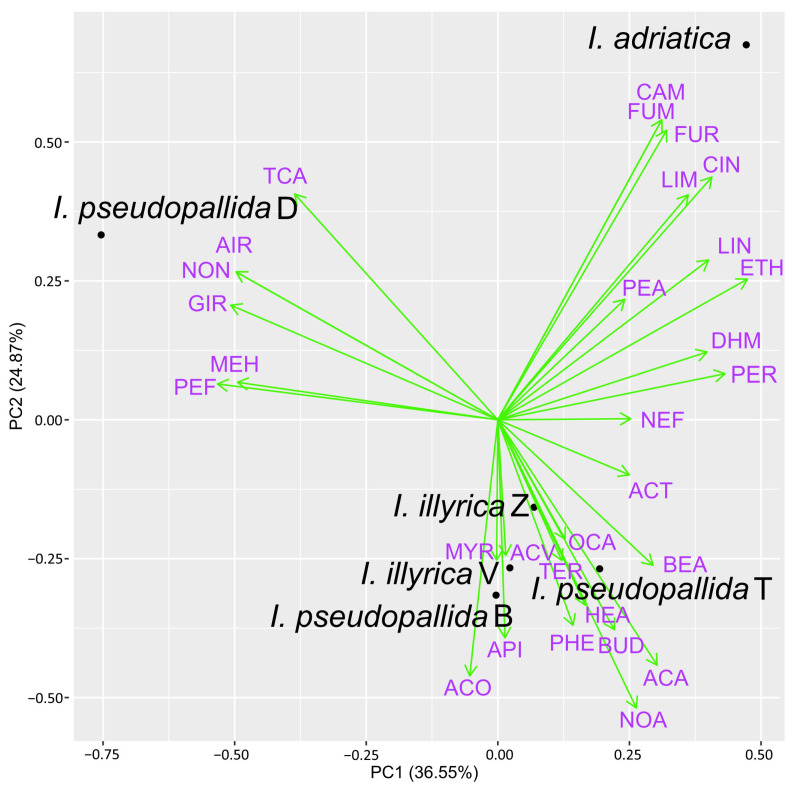
Biplot obtained by principal component analysis of the VOC composition of the six investigated *Iris* samples, based on their major components (average content ≥2.0% in at least one sample), detected using DVB/CAR/PDMS fiber; for compound abbreviation, cf. Supplementary Table S1.

**Figure 6:**
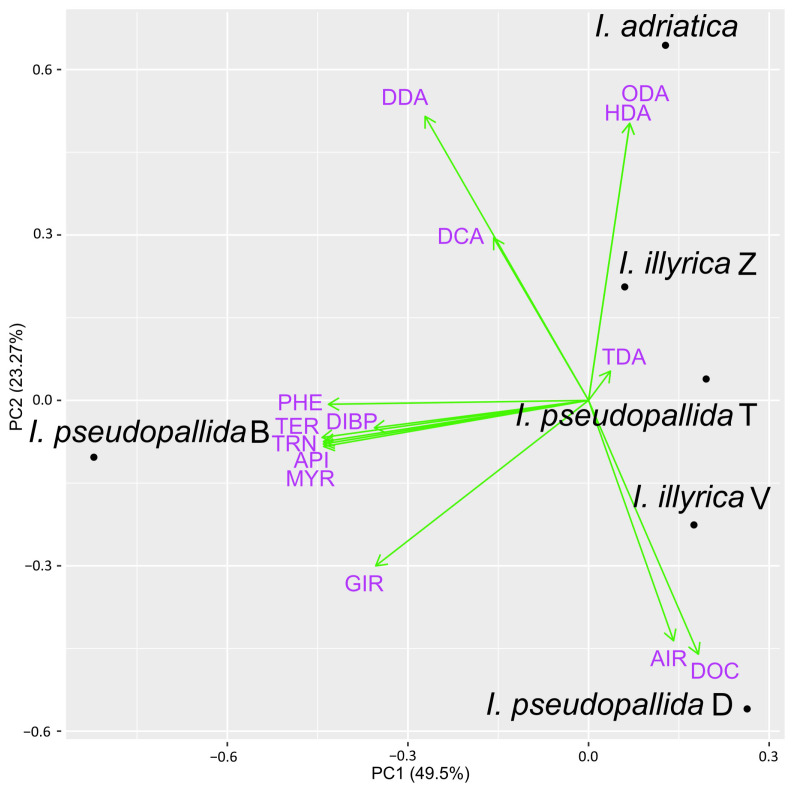
Biplot obtained by principal component analysis of EOs composition of the six investigated *Iris* samples, based on their major components (average content ≥2.0% in at least one sample), detected using HD; for compound abbreviation, cf. Supplementary Table S1.

Only those compounds having a content of at least 2.0% in one or more samples were further included in the PCA analyses, while the rest were considered to be of minor importance and were consequentially excluded from the analyses. In total, 30 major compounds were identified in the samples obtained by using PDMS/DVB fiber and DVB/CAR/PDMS fiber for HS–SPME (Figure 4 and Figure 5), while only 14 major compounds were identified in the EOs of the investigated *Iris* taxa (Figure 6).

The biplot constructed by the first two principal components showing the distribution of the investigated *Iris* samples and major VOCs detected with PDMS/DVB fiber is presented in Figure 4. Principal component 1 (PC1) accounted for 39.31%, and principal component 2 (PC2) for 21.10% of the total variance in the data. Clear separation from the remaining samples was observed for *I. adriatica*, with camphor, (furan-2-yl)methanol, limonene, and 1,8-cineole being the distinctive VOCs, as well as for *I. pseudopallida* D, with nonan-2-one, *cis*-α-irone, and *cis*-γ-irone as the distinctive VOCs. The remaining samples of *I. pseudopallida* and *I. illyrica* were characterized by a range of components (e.g., dihydromyrcenol, perilla aldehyde, benzyl alcohol, acetic acid, ethanol, and butan-2,3-diol) and were not further separated from each other.

The biplot constructed by the first two principal components showing the distribution of the investigated *Iris* samples and major VOCs identified with DVB/CAR/PDMS fiber is presented in Figure 5. PC1 accounted for 36.55%, and PC2 for 24.87% of the total variance in the data. As with the former fiber, clear separation based on the presence of the same characteristic compounds was obtained for *I. pseudopallida* D and *I. adriatica*, with furfural recognized as an additional distinguishing compound for the latter taxon. The remaining samples of *I. pseudopallida* and *I. illyrica* were grouped more closely together and were characterized primarily by acetic acid, α-copaene, and nonanal.

The biplot constructed by the first two principal components showing the distribution of investigated *Iris* samples and major EOs constituents is presented in Figure 6. PC1 accounted for 49.50%, and PC2 for 23.27% of the total variance in the data. As in the samples obtained by HS–SPME, *I. adriatica* was distinguished from other investigated taxa, based on the exclusive presence of two components that were not found in the extracts prepared by the former two methods, hexadecanoic acid and (*Z*,*Z*)-octadeca-9,12-dienoic acid. Moreover, *I. pseudopallida* D was not only again characterized by *cis*-α-irone but also by docosane. Unlike with the previous two methods, the PCA analysis of the samples VOCs obtained by HD resulted in additional separation of *I. pseudopallida* B. The latter sample was characterized by several monoterpene alcohols, such as terpinen-4-ol, α-terpineol, and myrtenol, together with α-pinene (monoterpene hydrocarbon) and 2-phenylethanol (aromatic alcohol). The remaining samples of *I. pseudopallida* and *I. illyrica* were grouped together.

## 3. Discussion

Out of the many *Iris* species that are known today, around 30 species have been reported for their usage in traditional medicine. Three of them, namely *I*. *pallida* (Dalmatian iris or sweet iris), *I. germanica* (German iris or blue German bearded iris), and *I. florentina* (Florentine iris or white German bearded iris), are extensively used by various industries, including the pharmacy, perfumery, cosmetic, and food industries [34,35], although other bearded irises could most likely be processed for the same purpose [36]. Solely in Italy, about 1000 tons of fresh iris rhizomes is used annually to produce iris EO (orris oil) [34]. According to the Expert Panel of the Flavor and Extract Manufacturers Association (FEMA) assessment, Orris Root Extract (FEMA 2830) and Orris Concrete Liquid Oil (FEMA 2829), which are obtained from the same three species, have an annual usage greater than or up to 1000 kg [37]. The abovementioned species belong to the group of bearded irises, which have multicellular caterpillar-like hairs on the external tepals of their flowers and are popular ornamental plants worldwide [25,35,38], as well as in Croatia, where the iris is not only a beloved garden flower but is also the Croatian national flower [39].

The main goal of the present study was to analyze the VOCs profiles obtained from rhizomes of three endemic bearded taxa from Croatia, *I. adriatica*, *I. illyrica*, and *I. pseudopallida*. A careful selection of extraction methods is key to target a particular class of VOCs. Degradation or polymerization of certain compounds and/or artifact formation are known to occur during conventional methods such as HD [40]. To gain a better insight into the VOCs that could be obtained from the studied species, their GC–MS profiles were compared after isolation by three different methods, two based on extraction of volatiles by HS–SPME using bipolar fibers comprising either two (PDMS/DVB) or three materials (DVB/CAR/PDMS), and the third one being the conventionally used HD. HD and steam distillation are, in general, the primary methods of choice for EO isolation [40]. As such, they have also been the most utilized methods in analyses of *Iris* rhizomes’ EO constituents [21,22,23,41,42,43,44].

In the present study, *Iris* EOs obtained by HD contained several long-chain fatty acids that were detected in greater abundance. As was expected, HD isolated more fatty acids in comparison to HS–SPME. The latter method extracted only short- and medium-chain fatty acids, such as acetic, caproic, and caprylic, as well as methyl and ethyl esters of caprylic acid. All EOs analyzed in the present study contained tetradecanoic (myristic) acid as (one of) the major constituent(s), while palmitic and linoleic acids were the major constituents found exclusively in *I. adriatica* EO. The latter taxon contained the least amount of myristic acid (4.20%). Myristic acid was especially high in *I. pseudopallida* B, *I. pseudopallida* T, and *I. illyrica* V and Z EO (31.92–97.01%). Myristic acid, followed by other long-chain fatty acids (e.g., lauric, capric, and palmitic acid) and/or their esters (e.g., palmitic, octadecanoic, or elaidic acid methyl ester), was found as the major constituent of several Syrian *Iris* species [22]. Lauric (1.18–3.90%) and capric acids (0.34–2.82%) were also detected in all studied samples, except for the irone-rich *I. pseudopallida* D, a result that is in accordance with the results obtained for the dried rhizomes of *I. florentina* EO [41]. In fact, the relative amounts of irones, which were found only in *I. pseudopallida* D and *I. pseudopallida* B, were inversely proportional to the amounts of myristic acid. Similarly, Kara et al. found that, after three months of storage, the relative percentage of myristic acid falls from 87.50% to 77.39% [41]. As already mentioned, orris oil, the EO of *Iris* species used for commercial purposes and which is usually obtained from rhizomes by steam distillation, is a cream-colored solid that is rich in myristic acid and other fatty acids [34]. It can be used as such or further processed to eliminate fatty acids [4].

Tetradecanoic acid was also found to be a significant component of EO obtained through microdistillation from rhizomes of *I. kerneriana* Asch. and Sint. ex Dykes [20]. Its presence was also confirmed in EO of *I. pallasii* Fisch. ex Trev. seeds, but in a much lower percentage than in our study (0.08–1.12%) [45]. On the contrary, the same study revealed the presence of linoleic acid ((*Z*,*Z*)-octadeca-9,12-dienoic acid), which was detected in higher percentages (50.36–65.35%) than in the *I. adriatica* EO investigated in the present study. Other studies have also reported the presence of (*Z*,*Z*)-octadeca-9,12-dienoic acid in some *Iris* species, such as *I. lactea* Pall. seed oil (41.31%) [46], leaf extracts of *I. germanica, I. pallida, I. variegata L.,* and *I. hungarica* Waldst. and Kit (1.8–7.2%) [47] and the rhizome extract of *I. carthaliniae* Fomin (6.05%) [48]. Hexadecanoic acid was also found in *I. planifolia* (Mill.) T. Durand and Schinz whole-plant EO [49], but in a lower percentage compared to the ones found in our study (18.50%). Other studies have also reported the presence of fatty acids in *Iris* EOs extracted by steam distillation, such as myristic, lauric, and capric acid, that were found in the EOs extracted from the rhizomes of *I. pallida* [42], along with a substantial amount of capric acid in *I. carthaliniae* rhizome EO [43]. Fatty acids such as myristic, lauric, and palmitic are important ingredients in cosmetic preparations, where they serve as emulsifiers, softeners, cleansers, or brighteners. Fatty acids, together with ceramides and cholesterol, are key intercellular lipid components of stratum corneum. As such, long-chain fatty acids are especially important for the maintenance of normal skin-barrier function, resistance against the entry of harmful chemicals, and the prevention of excessive transepidermal water loss (TEWL) [50].

It was suggested that, in the EOs of *I. pallida* and *I. germanica*, more than 90% of the GC–eluted pool may be accounted for by just seven compounds, including around 85% of myristic acid (and lauric acid) and 6–14% of irones, mainly the *cis*-α- and *cis*-γ-irones [3]. The same irone isomers were detected as the major irones in our study. The content of *cis*-α-irone established in *I. pseudopallida* D after HD was much higher (24.70%) than that reported above for *I. pallida* and *I. germanica* EOs, while the contents of *cis*-γ-irone found in the remaining two *I. pseudopallida* EOs were similar (4.48–8.43%) to those reported [3]. The contents of *cis*-α-irone observed in the present study were higher than the 2.71% reported for the EO extracted from *I. graminea* Thunb. leaves obtained by steam distillation [44]. Although irones are recognized as the most valuable components of orris oil [34], it is important to note that these compounds are not present in the original plant material (fresh *Iris* rhizomes), but they develop during its drying and storage. As rhizomes age, oxidative degradation of their precursors (iridals) results in the formation of irones [24], the compounds that give the characteristic violet-like fragrance to iris rhizome EOs [3]. Our results are in agreement with this, keeping in mind that *I. pseudopallida* D, which was the first collected and, therefore, the oldest analyzed sample of the named taxon, possessed a much greater amount of irones compared to the other sample of the same taxon collected about 20 days later (Table 4). This is a relatively short period of time in comparison to the time that *Iris* rhizomes collected for commercial purposes are usually being stored to produce significant amounts of irones, i.e., at least 2–3 years. For example, an analysis of the EO obtained from a 3-month-old rhizomes of a 3-year-old *I. florentina* resulted in merely 4.21% of α-irone and 7.88% of *trans*-2,6-γ-irone, while none was detected in the fresh rhizomes [41].

Keeping in mind that irone formation is generally considered to be a slow process lasting at least 2–3 years or longer [3], it is interesting to note that less than a month had passed between the collection of our first and last sample (Table 4). This indicates the possibility of a much faster conversion rate. However, the two remaining samples of *I. pseudopallida* were harvested on the same day from two separate locations, and irones were found only in one of them. Therefore, the difference in sample collection can only partially explain the observed variations in irone contents. Other factors may also have influenced the irone production, such as the environmental conditions in which the plants were growing and the age of rhizomes before harvest. Based on the literature data, it is possible to assume that the relatively short period of storage of the samples analyzed in the present study was not enough for most of them to develop detectable amounts of irones, but they may be found in samples stored for a longer period of time. It was beyond the aims of the present work to account for various factors that could influence the irone production and VOC compositions in general. A separate study that would include more samples and/or their analysis at various time points in the future could be beneficial, in combination with different harvesting periods, keeping in mind that, in the present study, rhizomes were collected in April, while for commercial purposes, they are usually collected during July and August [4]. It would probably be more difficult to collect a much greater number of samples for each taxon if the plant material would need to be collected from the wild and would need to include underground parts of endemic taxa, which are not always easy to find and/or are not sufficiently represented for sampling. Therefore, an analysis of different taxa cultivated in the same botanical garden might be a better and more sustainable approach, which could also eliminate environmental factors contributing to the synthesis of iridals (irone precursors).

Be that as it may, considering their commercial importance, it is of great interest to establish the amounts of irones in EOs obtained from *Iris* rhizomes. HS–SPME was previously shown to be suitable for irone extraction [24]. Our results also indicate that this method could be more suitable for irone detection than HD, considering that the samples extracted by HS–SPME contained almost double the amounts of irones compared to the ones extracted by the previous method, with the relative contents varying between 43.74% and 45.76% for *cis*-α-irone. However, the fibers used in the present study were not suitable for the extraction of long-chain fatty acids, which are the known major characteristic constituents of *Iris* EOs. Conversely, short-to-medium-chain (volatile) fatty acids such as acetic, caproic, and caprylic were found in these samples, with the major one being acetic acid (1.91–31.02%), which was not present in the irone-rich sample of *I. pseudopallida*. Short-chain fatty acids, including acetic and caproic acids, are important for different industries, including the pharmacy, cosmetic, and perfume industry [51]. However, these compounds were not detected in the EOs obtained by HD. The loss of volatile compounds and oxidative degradation are some of the possible drawbacks of HD [40]. With this in mind, HS–SPME may be more suitable for terpene extraction from the samples. For example, limonene, a monoterpene hydrocarbon which was found to be a major VOC characteristic of *I. adriatica* after using HS–SPME (and was also present in other samples), was not detected in the same taxon after HD. It can be observed from the obtained results that different conclusions could be gained about the studied samples depending on the method of choice being either HD or HS–SPME. This highlights the importance of combining different extraction methods in the analyses and comparison of VOCs from rhizomes of different *Iris* taxa. Different fiber coatings extract analytes from samples by either absorption (liquid coatings) or adsorption (solid coatings). The polar polyacrylate (PA) and non-polar single-phase polydimethylsiloxane (PDMS) fibers are included in the category of coatings based on absorption. The adsorption mechanism is used by mixed-phase bipolar fibers, such as PDMS/DVB, Carbowax^®^/divinylbenzene (CW/DVB), CAR/PDMS, and DVB/CAR/PDMS [52]. According to several studies, bipolar fiber coatings have the ability to extract a greater range of compounds than single coatings [53,54,55]. The DVB/CAR/PDMS and PDMS/DVB fibers, which have been demonstrated to be most appropriate for untargeted HS–SPME analysis of volatiles [32], were used in this study to analyze the VOCs of *I. pseudopallida*, *I. illyrica*, and *I. adriatica*.

In our study, comparable VOC profiles, in contrast to HD, were obtained using the said fibers, PDMS/DVB and DVB/CAR/PDMS, as previously reported [32]. However, more headspace VOCs were extracted using PDMS/DVB fiber (77) than using DVB/CAR/PDMS fiber (71), which is in accordance with the results reported by Mariano et al. [56], who explored *Eugenia klotzschiana* O. Berg fruit pulp and extracted 23 VOCs from PDMS/DVB fiber, 17 from PA fiber, and only 8 from DVB/CAR/PDMS fiber. When compared to each other, PDMS/DVB fiber extracted more monoterpene hydrocarbons, oxygenated monoterpenes, and sesquiterpene hydrocarbons, while DVB/CAR/PDMS extracted more fatty acids and related esters. Similarly, in our recent investigation of *Sideritis romana* L. and *S. montana* L. VOCs, it was also observed that PDMS/DVB fiber may extract more sesquiterpene hydrocarbons and oxygenated sesquiterpenes, and DVB/CAR/PDMS fiber extracted more oxygenated monoterpenes and other compounds (mostly non-terpenes) [57]. The affinity of a fiber for a particular VOC depends on the principle of “like dissolves like”, where PDMS/DVB fiber is more polar than the DVB/CAR/PDMS fiber and preferred for the extraction of analytes with higher molecular weights (MW 50–300) [32,58]. A similar trend was observed in our study, in which compounds of MWs higher than 100, such as acetovanillone, benzyl alcohol, camphor, *trans*-caryophyllene, 1,8-cineole, α-copaene, decanal, dihydromyrcenol, *cis*-α-irone, limonene, linalool, neryl formate, nonanal, perilla aldehyde, 2-phenylethanol, and undecan-2-one, were (in most cases) extracted in greater amounts using PDMS/DVB fiber. On the other hand, compounds with MWs up to 100 and/or with the lowest RIs (<900), such as acetic acid, acetoin, butan-2,3-diol, ethanol, furfural, and hexanal, were (in most cases) better extracted by the less polar DVB/CAR/PDMS fiber (Supplementary Table S1).

Considering that the present study is the first to report the VOC compositions of *I. pseudopallida*, *I. illyrica*, and *I. adriatica*, there is a lack of literature data to compare our results with other studies. However, some studies have reported VOC compositions of flowers of other *Iris* species [25,59]. For example, according to Yuan et al. [25], an HS–SPME/GC–MS analysis of floral scent profiles revealed that, out of 27 analyzed accessions of three bearded iris species, the alcohols constituted the most predominant components in 7 *I. germanica* and 2 *I. pumila* cultivars. The irises included in our study are also representatives of bearded irises (subgenus *Iris*, section *Iris*), which are characterized by bearded outer tepals. Hereby, *I. pseudopallida* and *I. illyrica* are closely related tall bearded taxa from the *I. pallida* complex, while *I. adriatica* is a dwarf bearded taxon from the *I. pumila* complex [27,60]. This fact may explain why, in the observed results of PCA analyses, the prior two taxa were often grouped together, while *I. adriatica* was separated from them.

The main individual compound identified in all investigated samples, except for *I. pseudopallida* D, which was extracted using HS–SPME, was perilla aldehyde. Perilla aldehyde is a compound characteristic of *Perilla frutescens* (L.) Britt. EO and one of the major contributors to its insecticidal and repellent activity [61]. This compound was better extracted using the PDMS/DVB fiber, same as some other aldehydes, such as nonanal and decanal, and sesquiterpene hydrocarbons such as *trans*-caryophyllene and α-copaene. Nonanal is another compound that was found in the same five samples. Nonyl aldehyde (nonanal) has been recently reported as a common compound in the floral scents of *I. uniflora* Pall. ex Link, *I. typhifolia* Kitag., and *I. sanguinea* Hornem., found after HS–SPME using DVB/CAR/PDMS fiber [59]. All investigated taxa also contained *trans*-caryophyllene. *trans*-Caryophyllene (2.86%) was one of the major sesquiterpene hydrocarbons of EO extracted from aerial fresh blooms of *I. nigricans* during the pre-flowering stage [29]. α-Copaene was also detected in all harvested samples of *I. pseudopallida* and *I. illyrica*, but not in *I. adriatica*, with the concentrations being greater (0.98–5.14% on both fibers) than what was previously reported for the EO extracted through steam distillation from rhizomes of *I. carthaliniae* and *I. medwedewii* Fomin (0.2% and 0.1%) [43]. One of the main individual compounds in all three *I. pseudopallida* populations and both *I*. *illyrica* populations (1.30–9.47% on both fibers), and which was also found in *I. adriatica*, was 6-methylhept-5-en-2-one. Based on previous findings, 6-methylhept-5-en-2-one can be produced during the oxidation reaction of lycopene [62]. Our results were similar to the results obtained for the EOs obtained by hydrodistillation from air-dried flowers and rhizomes of *I. persica* (7.1% and 11.4%) [21], and *I. pseudacorus* L. (11%) EOs, isolated from the flowers by microdistillation, as well as *I*. *kerneriana* flowers and rhizomes (7.1% and 7.7%) [20].

Limonene and 1,8-cineole (3.52–4.79% and 2.38–3.82%), along with furfural (1.80–7.91%), were among the primary individual compounds identified for *I. adriatica*. Limonene has been previously found in a lower percentage in EOs of other *Iris* species, such as *I. bulleyana* and *I. nigricans* rhizome EOs (1.65% and 2.02%) [23,29], whereas 1,8-cineole was found in a smaller percentage (2.0%) in *I. persica* rhizome EO [21]. Conversely, compared to *I. adriatica*, which was examined in our study, *I. persica* EOs from the flowers, leaves, rhizomes, and bulbs contained more furfural (13.8–39.0%) [21]. Furfural is a heterocyclic aldehyde that is probably formed during the hydrolysis of hemicellulose (depending on the type of biomass), and it can be used as a flavoring agent in the food industry, among other things [63]. Limonene and 1,8-cineole, with their lemon-like and eucalyptus aromas, are also recognized as flavoring compounds [64,65].

Deng et al. reported on the composition of Chinese EO obtained by hydrodistillation from rhizomes of *I. bulleyana* in which aristolone and cuparene were found to be the most abundant components [23]. In a study of the EO isolated from aerial fresh blooms and rhizomes of *I. nigricans*, at the pre-flowering stage, aliphatic hydrocarbons and their derivatives and oxygenated monoterpenes were the dominant compounds in the EO obtained from aerial parts, while at the post-flowering stage, as determined by GC–MS, aliphatic hydrocarbons and their derivatives prevailed. On the other hand, the EOs of rhizomes harvested after flowering were characterized by monoterpene hydrocarbons and oxygenated monoterpenes [29]. Since terpenes isolated by HD accounted for less than 10% of the total identified VOCs in five out of six of the studied samples, the HS–SPME method may generally be considered more suitable for their extraction from *Iris* samples. However, several oxygenated monoterpenes and monoterpene hydrocarbons, including terpinen-4-ol, α-terpineol, myrtenol, and α-pinene, were in fact detected in higher percentages in one sample of *I. pseudopallida* EO.

Considering the large number of VOCs that have been identified for the first time in this study, we believe that *I. pseudopallida*, *I. illyrica*, and *I. adriatica* show potential for additional research, which could provide evidence for their potential pharmaceutical and cosmetic applications, especially considering their observed irone and/or long-chain fatty acid contents.

## 4. Materials and Methods

### 4.1. Plant Material

Rhizomes of *Iris pseudopallida*, *I. illyrica,* and *I. adriatica* were collected during April 2019 from six different natural populations in Croatia (Table 4). The voucher specimens were deposited at the herbariums of the Department of Biology, Faculty of Science; and the Department of Pharmaceutical Botany, Faculty of Pharmacy and Biochemistry, University of Zagreb, Croatia. After rinsing with water, samples were left to dry in shade at room temperature and were afterwards subjected to HS–SPME and HD, followed by GC–MS analyses within one month from harvest of the samples that were last collected.

### 4.2. Solid-Phase Microextraction (SPME) Fibers and Extraction Procedure

Headspace solid-phase microextraction (HS–SPME) was performed on a manual SPME holder, using the divinylbenzene/carboxene/polydimethylsiloxane (DVB/CAR/PDMS) and polydimethylsiloxane/divinylbenzene (PDMS/DVB) fibers that had been conditioned according to Supelco CO.’s (Bellefonte, PA, USA) instructions before extraction. Separately, 1 g of finely cut samples were placed in glass vials (5 mL) and hermetically sealed with PTFE/silicone septa. During equilibration (15 min) and extraction by HS–SPME (45 min), the vials were kept in a water bath (60 °C). After extraction, the SPME fiber was removed and inserted into the GC–MS injector (250 °C) for thermal desorption (6 min). This procedure was similar to the ones previously described [57,66]. HS–SPME was performed in duplicate, and the results are expressed as mean values of percent composition (Table 1 and Table 2).

### 4.3. Hydrodistillation (HD)

A modified Clevenger apparatus was used for hydrodistillation (HD), lasting for 2 h, using 1 mL of the solvent trap (pentane/diethyl ether 1:2 *v/v*). The 10 g of prepared samples were cut into small pieces and used separately for hydrodistillation. The EO that had dissolved in the solvent trap was removed using a pipette, and then it was carefully concentrated by slowly flowing nitrogen until a volume of 0.2 mL was reached and dried through the layer of MgSO_4_ in a small glass funnel. Every sample went through a duplicate hydrodistillation. The volume used for GC–MS analysis was 1 µL.

### 4.4. GC–MS Analysis

Gas chromatography–mass spectroscopy (GC–MS) analysis was conducted using a gas chromatograph model 7820a (Agilent Technologies, Palo Alto, CA, USA) that was equipped with a mass selective detector (MDS) model 5977E (Agilent Technologies, Palo Alto, CA, USA) and a HP-5MS capillary column (5% phenylmethylpolysiloxane, Agilent J and W; 30 m × 0.25 mm i.d., coating thickness of 0.25 μm). Helium was used as the carrier gas (He 1.0 mL/min). The oven temperature was set at 70 °C for 2 min and then increased to 200 °C at a rate of 3 °C/min and held at 200 °C for 15 min. The MSD (EI mode) was used at 70 eV, with a mass range of 30–300 amu [66].

The compounds’ identification was based on the retention indices (RIs) that were calculated by comparing the retention times of the *n*-alkanes (C_9_–C_25_) to data from the literature (National Institute of Standards and Technology, Gaithersburg, MD, USA) and the mass spectra of the components, which corresponded to those of the mass spectral libraries Wiley 9 (Wiley, New York, NY, USA) and NIST 17 (Gaithersburg, MD, USA). The normalization method (without correction factors) was used to calculate the percentage composition. Table 1 and Table 2 show the average component percentages calculated from the results obtained from two replicate GC–MS analyses [66].

### 4.5. Principal Component Analysis

Principal component analysis (PCA) was conducted on the volatile constituents with an average relative percentage ≥2.0% in at least one of the samples obtained by using a particular method of sample preparation, i.e., HS–SPME using divinylbenzene/carboxene/polydimethylsiloxane (DVB/CAR/PDMS) fiber, HS–SPME using polydimethylsiloxane/divinylbenzene (PDMS/DVB) fiber, or HD. In total, in order to examine the interrelationships among the three investigated populations of *I. pseudopallida*, two investigated populations of *I. illyrica* and one investigated population of *I. adriatica*, three analyses were performed using the function prcomp in R version 4.4.1. [67]. Scaling was set to “TRUE” in order to perform the analysis on normalized data. Six observation of 30 variables (contents of major components) recorded for the samples obtained by using PDMS/DVB fiber, six observations of 30 variables recorded for the samples obtained by using DVB/CAR/PDMS fiber, and six observations of 14 variables recorded for the samples obtained by HD were plotted using the function autoplot from the package ggfortify [68,69].

## 5. Conclusions

This is the first study to report on the VOC profiles of *Iris pseudopallida*, *I. illyrica*, and *I. adriatica*. Samples prepared from the plant material collected from six different locations in Croatia, three for *I. pseudopallida*, two for *I. illyrica*, and one for *I. adriatica*, were analyzed by HD and HS–SPME/GC–MS (using PDMS/DVB and DVB/CAR/PDMS fibers). A total of 73 constituents were identified in the samples obtained by HD, while 77 VOCs were extracted on PDMS/DVB fiber and 71 on DVB/CAR/PDMS fiber. HD extracted more fatty acids and fatty acid esters, of which tetradecanoic (myristic) acid, (*Z*,*Z*)-octadeca-9,12-dienoic (linoleic) acid, and hexadecanoic (palmitic) acid were the major ones. Myristic acid was found to be a major component of EOs from all investigated taxa, while palmitic and linoleic acids were characteristic for *I. adriatica* EO. Lauric and capric acids were also noted in five out of six samples. In two out of the three studied *I. pseudopallida* EOs, *cis*-α- and/or *cis*-γ-irone were detected as the major constituents. One of these (the one with the higher irone content), as well as one *I. illyrica* EO, were especially rich in docosane (cca. 45–55% content), while the other was characterized by the presence of monoterpene alcohols (myrtenol, terpinen-4-ol, and α-terpineol) and the monoterpene hydrocarbon α-pinene.

The most abundant VOCs detected by HS–SPME/GC–MS on both fibers for two out of the three investigated *I. pseudopallida* plants were oxygenated monoterpenes, sesquiterpene hydrocarbons, and aliphatic and aromatic alcohols, while the third (the oldest) one contained mainly norisoprenoids, with *cis*-α-irone and *cis*-γ-irone making >50% of total VOCs constituents. As for the investigated sample of *I. adriatica* and the two investigated *I. illyrica* samples, aliphatic and aromatic alcohols were the predominant compounds found using both fibers. The most abundant individual VOCs determined in our study by HS–SPME/GC–MS were perilla aldehyde, acetic acid, 6-methylhept-5-en-2-one, *cis*-α-irone, *cis*-γ-irone, butan-2,3-diol, *trans*-caryophyllene, furfural, ethanol, benzyl alcohol, myrtenol, α-copaene, hexanal, nonanal, nonan-2-one, and acetovanillone. Out of these, 6-methylhept-5-en-2-one, *trans*-caryophyllene, and ethanol were common for all studied samples. Perilla aldehyde, butan-2,3-diol, acetic acid, 2-phenylethanol, benzyl alcohol, hexanal, and nonanal were found in all studied samples except for the irone-rich *I. pseudopallida* (>50% irones). This sample was characterized by both *cis*-α-irone (as the major compound) and *cis*-γ-irone, while the latter irone isomer was the only irone present in an additional sample of *I. pseudopallida*. The former sample of *I. pseudopallida* was also characterized by nonan-2-one. α-Copaene was a major compound in *I. pseudopallida* and *I. illyrica* but it was not found in *I. adriatica*. The latter taxon was, in turn, characterized by the presence of (furan-2-yl)methanol, camphor, limonene, and a high percentage of furfural. Furfural was also present in one sample of *I. illyrica*, the same that was characterized by acetovanillone. Furfural, as well as other compounds with MWs up to 100 and/or with the lowest RIs (<900), such as acetic acid, was better extracted by the less polar DVB/CAR/PDMS fiber, while PDMS/DVB fiber extracted greater amounts of compounds with higher MWs (>100).

Further extensive research of *I. pseudopallida*, *I. illyrica*, and *I. adriatica* could provide valuable information on their response to different environmental conditions, as well as the possibility of using the mentioned compounds in pharmacy and the cosmetic industry or perfumery. The most prominent compounds of investigated *Iris* rhizome EOs are comparable to the major ones commonly reported for commercial orris oil obtained from either *I. pallida*, *I. germanica*, or *I. florentina*, such as myristic acid, lauric acid, capric acid, *cis*-α-irone, and *cis*-γ-irone. Since the production of irones, the most valuable constituents of orris oil, is usually believed to be a slow process lasting at least 2–3 years or longer, their detection in a relatively short period after harvesting (<2 months) is interesting from the perspective of the potential utilization of the investigated taxa, especially the *I. pseudopallida* taxon. Future studies could include more samples of the studied taxa and/or different harvesting periods (e.g., pre-flowering, flowering, and post-flowering), as well as analyses of VOC and EO constituents in different time points after the collection of plant material (e.g., within one week, one month, three months, six months, and one and two years from harvest). Inclusion of additional taxa and a greater number of samples for each of them might also be interesting from a phytotaxonomic perspective, especially if they could be harvested from a common location, such as a (botanical) garden, in order to eliminate the possible influence of environmental factors.

## Figures and Tables

**Table 1:** Volatile organic compound (VOC) composition (%) of *I. pseu dopallida*, *I. illyrica*, and *I. adriatica* obtained by headspace solid-phase microextraction (HS–SPME) using polydimethylsiloxane/divinylbenzene (PDMS/DVB) fiber (and analyzed by GC–MS).

No.	Compound	RI	RI_L_	RI_L_ Reference	SI	*I. pseudopallida* B	*I. pseudopallida* D	*I. pseudopallida* T	*I. illyrica* V	*I. illyrica* Z	*I. adriatica*
Aliphatic and Aromatic Alcohols and Phenols											
1	Ethanol	<900	448	[33]	97%	2.21	1.39	6.86	4.79	3.65	2.66
5	Pentan-1-ol	<900	779	[33]	98%	0.41	-	-	-	-	0.93
6	Butan-2,3-diol	<900	802	[33]	97%	1.78	-	6.38	5.29	10.27	2.18
9	(Furan-2-yl)methanol	<900	864	[33]	96%	-	-	-	-	-	2.75
10	Hexan-1-ol	<900	867	[33]	96%	0.18	-	0.38	0.73	1.14	1.09
11	2-Butoxyethanol	912	912	[33]	98%	0.24	-	-	0.58	0.75	0.95
14	1-Butoxypropan-2-ol	949	945	[33]	95%	0.34	-	-	0.31	1.27	1.39
17	Phenol	986	987	[33]	99%	-	-	0.20	0.36	0.36	0.53
24	2-Ethylhexan-1-ol	1035	1034	[33]	97%	0.85	-	0.40	0.49	1.82	1.66
27	Benzyl alcohol	1042	1042	[33]	99%	2.03	-	5.46	3.40	2.46	2.40
33	Octan-1-ol	1076	1076	[33]	98%	0.50	-	0.35	-	0.34	0.47
38	Nonan-2-ol	1103	1102	[33]	96%	-	0.50	-	-	-	-
41	2-Phenylethanol	1120	1120	[33]	99%	4.67	-	1.76	1.70	4.76	0.90
69	Dodecan-1-ol	1479	1478	[33]	97%	-	-	1.40	-	-	-
	Total identified (%)					13.21	1.89	23.19	17.65	26.82	17.91
Fatty Acids and Fatty Acid Esters											
2	Acetic acid	<900	600	[33]	97%	2.88	-	6.29	6.95	1.91	3.74
16	Hexanoic (caproic) acid	979	977	[33]	98%	0.37	-	0.50	0.65	-	-
42	Methyl octanoate	1131	1127	[33]	97%	-	-	1.00	-	-	-
46	Octanoic (caprylic) acid	1181	1180	[33]	97%	-	-	2.15	-	-	-
49	Ethyl octanoate	1198	1196	[33]	98%	-	-	1.05	-	0.36	-
63	Methyl decanoate	1330	1328	[33]	98%	0.26	-	0.57	-	-	-
	Total identified (%)					3.51	-	11.56	7.60	2.27	3.74
Aliphatic and Aromatic Aldehydes											
3	Pentanal	<900	698	[33]	98%	-	3.28	-	2.48	-	-
7	Hexanal	<900	799	[33]	97%	0.81	-	0.71	2.57	1.14	0.96
8	Furfural	<900	848	[33]	98%	-	-	-	0.31	0.34	1.80
15	Benzaldehyde	971	972	[33]	99%	0.68	-	1.08	0.87	0.85	1.31
22	1*H*-Pyrrole-2-carboxaldehyde	1014	1015	[33]	95%	-	-	-	-	-	1.10
28	Phenylacetaldehyde	1052	1051	[33]	98%	-	1.16	-	-	-	0.56
29	1-Ethyl-2-formyl pyrrole	1058	1046	[33]	95%	-	-	-	-	-	0.82
40	Nonanal	1109	1108	[33]	98%	2.78	-	3.25	3.55	3.10	1.22
53	Decanal	1210	1210	[33]	99%	0.62	-	2.35	-	0.74	0.49
62	Undecanal	1311	1309	[33]	98%	-	-	0.76	-	-	-
66	Dodecanal	1413	1412	[33]	97%	-	-	1.01	-	-	-
	Total identified (%)					4.89	4.44	9.16	9.78	6.17	8.26
Aliphatic and Aromatic Ketones											
4	Acetoin	<900	720	[33]	98%	-	-	2.84	2.86	1.39	1.04
19	6-Methylhept-5-en-2-one	992	991	[33]	99%	1.98	3.59	1.61	3.21	9.47	1.33
31	2-Acetylpyrrole	1068	1065	[33]	96%	-	-	0.29	-	-	-
32	Acetophenone	1074	1072	[33]	96%	0.16	-	-	-	-	0.06
36	Nonan-2-one	1091	1091	[33]	98%	-	4.99	-	-	-	-
60	Undecan-2-one	1297	1296	[33]	97%	0.43	2.01	1.28	1.60	0.43	0.13
70	Acetovanillone	1491	1491	[33]	97%	0.11	-	-	7.12	-	-
	Total identified (%)					2.68	10.59	6.02	14.79	11.29	2.56
Lactone											
12	γ-Butyrolactone	922	925	[33]	96%	0.24	-	0.98	0.70	0.92	0.85
Monoterpene Hydrocarbons											
13	α-Pinene	945	942	[33]	98%	4.48	-	0.20	2.40	1.11	-
18	β-Pinene	986	985	[33]	98%	0.75	-	-	-	-	-
20	β-Myrcene	996	997	[33]	97%	-	-	0.50	-	-	0.65
23	*p*-Cymene	1033	1030	[33]	98%	0.85	0.18	-	0.27	0.46	0.37
25	Limonene	1037	1035	[33]	98%	1.46	-	0.90	0.88	0.57	4.79
30	γ-Terpinene	1067	1064	[33]	99%	-	0.42	-	-	-	-
	Total identified (%)					7.54	0.60	1.60	3.55	2.14	5.81
Furan											
21	2-Pentylfuran	997	998	[33]	97%	0.53	0.84	-	0.58	1.35	-
Oxygenated Monoterpenes											
26	1,8-Cineole	1041	1037	[33]	98%	0.68	-	2.65	0.34	1.16	3.82
34	Dihydromyrcenol	1078	1075	[33]	96%	0.95	-	3.03	1.60	3.46	2.45
35	*trans*-Linalool oxide	1080	1081	[33]	96%	0.27	-	-	-	-	-
37	6-Camphenone	1101	1095	[33]	95%	0.43	-	-	-	-	-
39	Linalool	1104	1102	[33]	98%	1.83	-	1.91	0.75	1.64	2.57
43	*trans*-Pinocarveol	1147	1147	[33]	97%	0.85	-	-	-	-	-
44	Camphor	1152	1149	[33]	99%	-	-	-	-	-	3.25
45	Borneol	1173	1172	[33]	99%	0.53	-	0.22	-	-	0.47
47	Terpinen-4-ol	1184	1184	[33]	98%	0.33	-	0.50	-	-	0.51
48	α-Terpineol	1196	1195	[33]	98%	3.19	-	1.53	0.66	-	0.76
50	Myrtenol	1199	1198	[33]	97%	6.33	-	-	-	-	-
54	β-Citronellol	1234	1232	[33]	97%	-	-	1.35	-	-	-
55	Carvacrol methyl ether	1241	1246	[33]	96%	0.72	-	-	-	-	-
56	Neryl formate	1262	1261	[33]	96%	-	-	4.02	-	-	1.20
57	(*E*)-Citral	1276	1278	[33]	96%	0.96	-	1.43	-	-	-
58	Perilla aldehyde	1279	1279	[33]	97%	20.55	-	17.76	19.72	30.00	26.83
59	Bornyl acetate	1290	1288	[33]	98%	0.38	-	-	-	-	1.38
	Total identified (%)					38.00	-	34.40	23.07	36.26	43.24
Alkanes											
51	Dodecane	1200	1200	[33]	98%	-	-	1.72	-	-	-
61	Tridecane	1300	1300	[33]	97%	-	-	1.00	-	-	-
65	Tetradecane	1400	1400	[33]	98%	0.34	-	1.20	0.25	0.25	0.52
72	Pentadecane	1500	1500	[33]	97%	-	-	-	-	-	0.52
	Total identified (%)					0.34	-	3.92	0.25	0.25	1.04
Norisoprenoids											
52	Safranal	1204	1205	[33]	98%	1.46	-	-	-	1.69	-
75	*cis*-α-Irone	1544	1546	[33]	97%	-	45.76	-	-	-	-
76	*cis*-γ-Irone *	1551	-	[24]	95%	1.87	7.17	-	-	-	-
	Total identified (%)					3.33	52.93	-	-	1.69	-
Sesquiterpene Hydrocarbons											
64	α-Copaene	1381	1376	[33]	96%	5.14	1.21	2.02	3.62	2.61	-
67	*trans*-Caryophyllene	1424	1423	[33]	97%	3.40	7.24	1.51	1.97	2.67	4.51
68	α-Humulene	1459	1459	[33]	98%	0.73	0.27	0.42	0.66	0.80	1.13
71	α-Farnesene	1499	1503	[33]	97%	0.81	1.09	1.19	1.99	0.98	1.94
73	α-Muurolene	1504	1505	[33]	96%	0.62	1.73	-	-	-	-
74	δ-Cadinene	1520	1519	[33]	98%	-	1.17	-	-	-	-
	Total identified (%)					10.70	12.71	5.14	8.24	7.06	7.58
Aromatic Ester											
77	Benzyl benzoate	1769	1770	[33]	97%	-	-	0.23	-	-	-
	Total amount of identified compounds (%)					84.97	84.00	96.20	86.21	96.22	90.99

*—Tentatively identified based on the mass spectrum; RI—calculated (experimental) retention index; RI_L_—retention index from the literature (references); SI—selectivity index (the highest probability of the experimental mass spectrum matching the mass spectrum present in the reference library).

**Table 2:** Volatile organic compound (VOC) composition (%) of *I. pseudopallida*, *I. illyrica*, and *I. adriatica* obtained by headspace solid-phase microextraction (HS–SPME) using divinylbenzene/carboxene/polydimethylsiloxane (DVB/CAR/PDMS) fiber (and analyzed by GC–MS).

No.	Compound	RI	RI_L_	RI_L_ Reference	SI	*I. pseudopallida* B	*I. pseudopallida* D	*I. pseudopallida* T	*I. illyrica* V	*I. illyrica* Z	*I. adriatica*
Aliphatic and Aromatic Alcohols and Phenols											
1	Ethanol	<900	448	[33]	97%	4.75	3.22	4.70	5.23	4.34	7.01
5	Pentan-1-ol	<900	779	[33]	97%	-	-	-	1.70	-	0.82
6	Butan-2,3-diol	<900	802	[33]	97%	2.41	-	7.05	7.83	11.11	2.43
9	(Furan-2-yl)methanol	<900	864	[33]	96%	-	-	-	-	-	3.08
10	Hexan-1-ol	<900	867	[33]	96%	0.96	-	0.14	1.29	0.30	0.89
11	2-Butoxyethanol	912	912	[33]	97%	0.43	-	0.18	-	0.42	0.38
14	1-Butoxypropan-2-ol	949	945	[33]	95%	0.29	-	-	-	0.81	0.80
17	Phenol	986	987	[33]	99%	0.58	-	-	-	-	-
23	2-Ethylhexan-1-ol	1035	1034	[33]	97%	0.82	-	-	-	1.13	1.36
26	Benzyl alcohol	1042	1042	[33]	99%	1.52	-	5.08	1.58	1.23	1.62
30	Octan-1-ol	1076	1076	[33]	98%	0.59	-	0.28	-	-	0.27
35	Nonan-2-ol	1103	1102	[33]	98%	-	0.51	-	-	-	-
38	2-Phenylethanol	1120	1120	[33]	99%	4.31	-	1.38	0.77	3.36	0.83
65	Dodecan-1-ol	1479	1478	[33]	97%	-	-	0.93	-	-	-
	Total identified (%)					16.66	3.73	19.74	18.40	22.70	19.49
Fatty Acids and Fatty Acid Esters											
2	Acetic acid	<900	600	[33]	96%	14.29	-	29.76	29.20	31.02	12.00
16	Hexanoic (caproic) acid	979	977	[33]	98%	0.52	-	0.67	1.64	-	-
39	Methyl octanoate	1131	1127	[33]	97%	-	0.43	1.27	-	-	-
42	Octanoic (caprylic) acid	1181	1180	[33]	96%	-	-	2.35	-	-	-
45	Ethyl octanoate	1198	1196	[33]	97%	-	-	0.61	-	-	-
58	Methyl decanoate	1330	1328	[33]	98%	0.30	0.51	0.67	-	-	-
	Total identified (%)					15.11	0.94	35.33	30.84	31.02	12.00
Aliphatic and Aromatic Aldehydes											
3	Pentanal	<900	698	[33]	98%	-	-	-	2.65	-	2.31
7	Hexanal	<900	799	[33]	97%	1.46	-	1.49	4.67	1.30	1.04
8	Furfural	<900	848	[33]	98%	-	-	-	1.03	0.11	7.91
15	Benzaldehyde	971	972	[33]	99%	0.93	0.67	1.11	0.88	0.68	1.01
21	1*H*-Pyrrole-2-carboxaldehyde	1014	1015	[33]	96%	-	-	-	-	-	0.51
27	Phenylacetaldehyde	1052	1051	[33]	98%	-	0.99	-	-	-	0.38
28	1-Ethyl-2-formyl pyrrole	1058	1046	[33]	95%	-	-	-	-	-	0.92
37	Nonanal	1109	1108	[33]	97%	2.78	-	2.71	2.07	1.24	0.90
49	Decanal	1210	1210	[33]	99%	0.50	-	1.46	0.17	-	0.36
61	Dodecanal	1413	1412	[33]	98%	-	-	0.58	-	-	-
	Total identified (%)					5.67	1.66	7.35	11.47	3.33	15.34
Aliphatic and Aromatic Ketones											
4	Acetoin	<900	720	[33]	98%	-	-	2.01	2.42	5.27	2.11
18	6-Methylhept-5-en-2-one	992	991	[33]	99%	2.61	9.10	1.30	3.14	5.83	0.81
29	Acetophenone	1074	1072	[33]	97%	0.11	-	-	-	-	-
33	Nonan-2-one	1091	1091	[33]	96%	-	5.11	-	-	-	-
56	Undecan-2-one	1297	1296	[33]	97%	0.21	1.61	-	0.63	-	0.12
66	Acetovanillone	1491	1491	[33]	98%	0.41	-	-	3.85	-	-
	Total identified (%)					3.34	15.82	3.31	10.04	11.10	3.04
Lactone											
12	γ-Butyrolactone	922	925	[33]	96%	0.41	-	0.91	1.34	0.89	0.24
Monoterpene Hydrocarbons											
13	α-Pinene	945	942	[33]	96%	2.46	-	-	1.55	0.37	-
19	β-Myrcene	996	997	[33]	97%	-	-	-	-	-	0.69
22	*p*-Cymene	1033	1030	[33]	98%	0.68	0.67	-	-	0.17	-
24	Limonene	1037	1035	[33]	97%	1.40	-	0.42	0.60	0.20	3.52
	Total identified (%)					4.54	0.67	0.42	2.15	0.74	4.21
Furan											
20	2-Pentylfuran	997	998	[33]	96%	0.80	2.37	-	0.96	0.69	-
Oxygenated Monoterpenes											
25	1,8-Cineole	1041	1037	[33]	98%	0.12	-	0.56	0.57	0.75	2.38
31	Dihydromyrcenol	1078	1075	[33]	96%	1.06	-	1.42	-	2.09	2.07
32	*trans*-Linalool oxide	1080	1081	[33]	96%	0.30	-	-	-	-	-
34	6-Camphenone	1101	1095	[33]	95%	0.30	-	-	-	-	-
36	Linalool	1104	1102	[33]	98%	1.31	-	0.79	-	0.79	2.20
40	Camphor	1152	1149	[33]	99%	-	-	-	-	-	2.98
41	Borneol	1173	1172	[33]	98%	0.76	-	0.17	-	-	0.49
43	Terpinen-4-ol	1184	1184	[33]	97%	0.41	0.39	-	-	-	0.56
44	α-Terpineol	1196	1195	[33]	98%	3.01	-	1.14	0.25	-	0.64
46	Myrtenol	1199	1198	[33]	97%	6.04	-	-	-	-	-
50	β-Citronellol	1234	1232	[33]	97%	-	-	1.21	-	-	-
51	Carvacrol methyl ether	1241	1246	[33]	96%	0.59	-	-	-	-	-
52	Neryl formate	1262	1261	[33]	96%	-	-	2.52	-	-	1.01
53	(*E*)-Citral	1276	1278	[33]	95%	0.82	-	0.52	-	-	0.67
54	Perilla aldehyde	1279	1279	[33]	98%	15.63	-	8.09	6.47	17.59	20.08
55	Bornyl acetate	1290	1288	[33]	97%	0.27	-	-	-	-	1.24
	Total identified (%)					30.62	0.39	16.42	7.29	21.22	34.32
Alkanes											
47	Dodecane	1200	1200	[33]	98%	-	-	1.37	-	-	-
57	Tridecane	1300	1300	[33]	99%	-	-	0.58	-	-	-
60	Tetradecane	1400	1400	[33]	98%	0.05	-	0.71	-	-	0.35
	Total identified (%)					0.05	-	2.66	-	-	0.35
Norisoprenoids											
48	Safranal	1204	1205	[33]	98%	1.72	-	-	-	-	-
70	*cis*-α-Irone	1544	1546	[33]	96%	-	43.74	-	-	-	-
71	*cis*-γ-Irone *	1551	-	[24]	95%	1.99	7.87	-	-	-	-
	Total identified (%)					3.71	51.61	-	-	-	-
Sesquiterpene Hydrocarbons											
59	α-Copaene	1381	1376	[33]	96%	5.06	0.98	1.32	2.08	1.62	-
62	*trans*-Caryophyllene	1424	1423	[33]	97%	2.70	5.50	0.61	0.82	1.37	2.79
63	*trans*-α-Bergamotene	1441	1441	[33]	96%	-	-	-	-	-	0.15
64	α-Humulene	1459	1459	[33]	97%	0.59	0.05	0.32	0.28	0.22	0.73
67	α-Farnesene	1499	1503	[33]	97%	0.33	-	-	-	-	0.83
68	α-Muurolene	1504	1505	[33]	95%	0.39	1.41	-	-	-	-
69	δ-Cadinene	1520	1519	[33]	98%	-	0.86	-	-	-	-
	Total identified (%)					9.07	8.80	2.25	3.18	3.21	4.50
	Total amount of identified compounds (%)					89.98	85.99	88.39	85.67	94.90	93.49

*—Tentatively identified based on the mass spectrum; RI—calculated (experimental) retention index; RI_L_—retention index from the literature (references); SI—selectivity index (the highest probability of the experimental mass spectrum matching the mass spectrum present in the reference library).

**Table 3:** Essential oil (EO) composition (%) of *I. pseudopallida*, *I. illyrica*, and *I. adriatica* obtained by HD (and analyzed by GC–MS).

No.	Compound	RI	RI_L_	RI_L_ Reference	SI	*I. pseudopallida* B	*I. pseudopallida* D	*I. pseudopallida* T	*I. illyrica* V	*I. illyrica* Z	*I. adriatica*
Aliphatic and Aromatic Aldehydes											
1	Furfural	<900	848	[33]	98%	1.07	0.05	-	0.01	0.06	0.02
4	Heptanal	<900	894	[33]	98%	-	0.08	-	-	0.02	0.02
7	Benzaldehyde	971	972	[33]	99%	-	0.27	0.02	0.02	0.08	0.05
13	Octanal	1005	1004	[33]	97%	-	0.12	-	-	-	0.02
19	Phenylacetaldehyde	1052	1051	[33]	98%	0.98	0.26	0.02	0.02	0.14	0.10
21	2,6-Dimethylhept-5-enal	1059	1060	[33]	97%	1.01	0.14	-	-	0.17	-
29	Nonanal	1109	1108	[33]	96%	-	-	-	0.03	0.06	0.10
33	(*E*)-Non-2-enal	1166	1161	[33]	97%	-	-	-	0.02	0.05	-
41	Decanal	1210	1210	[33]	99%	-	0.09	-	-	0.05	0.04
47	Undecanal	1311	1309	[33]	98%	-	-	-	-	-	0.03
54	Dodecanal	1413	1412	[33]	97%	-	1.28	-	-	0.20	0.30
	Total identified (%)					3.06	2.29	0.04	0.10	0.83	0.68
Alkanes											
2	4-Methyloctane	<900	864	[33]	95%	-	0.05	-	-	-	-
46	Tridecane	1300	1300	[33]	98%	-	-	-	-	-	0.05
59	Pentadecane	1500	1500	[33]	97%	-	-	-	-	0.06	0.02
70	Heneicosane	2100	1600	[33]	98%	-	1.06	0.09	0.09	0.40	0.48
72	Docosane	2200	2200	[33]	97%	-	45.79	-	55.45	1.04	0.21
73	Tricosane	2300	2300	[33]	98%	0.96	1.66	0.10	0.20	1.86	1.84
	Total identified (%)					0.96	48.56	0.19	55.74	3.36	2.60
Aliphatic and Aromatic Alcohols and Phenols											
3	Hexan-1-ol	<900	867	[33]	96%	-	-	-	0.01	-	0.02
6	1-Butoxypropan-2-ol	949	945	[33]	95%	-	-	-	-	0.02	0.01
9	Phenol	986	987	[33]	99%	-	-	-	0.02	0.04	-
15	2-Ethylhexan-1-ol	1035	1034	[33]	97%	-	-	-	-	0.02	0.03
18	Benzyl alcohol	1042	1042	[33]	99%	-	0.09	-	-	-	0.04
24	Octan-1-ol	1076	1076	[33]	98%	-	0.11	-	0.01	0.02	0.04
27	2-Methoxyphenol	1093	1092	[33]	98%	-	0.11	-	-	0.07	0.03
31	2-Phenylethanol	1120	1120	[33]	99%	2.20	-	-	0.02	0.81	0.07
35	Nonan-1-ol	1176	1175	[33]	95%	-	-	-	-	-	0.05
48	2-Methoxy-4-vinylphenol	1318	1317	[33]	99%	0.96	0.12	0.02	0.08	0.33	0.08
57	Dodecan-1-ol	1479	1478	[33]	97%	-	0.07	-	-	0.13	0.30
	Total identified (%)					3.16	0.50	0.02	0.14	1.44	0.70
Monoterpene Hydrocarbons											
5	α-Pinene	945	942	[33]	98%	5.98	-	0.01	0.16	0.08	-
10	β-Pinene	986	985	[33]	97%	0.80	-	-	-	-	-
14	*p*-Cymene	1033	1030	[33]	98%	0.50	-	-	0.01	0.02	-
16	Limonene	1037	1035	[33]	97%	-	-	-	0.01	0.02	-
20	(*E*)-β-ocymene	1055	1054	[33]	98%	-	-	-	-	-	-
22	γ-Terpinene	1067	1064	[33]	99%	1.18	-	-	-	-	-
	Total identified (%)					8.46	-	0.01	0.18	0.12	-
Fatty Acids and Fatty Acid Esters											
8	Hexanoic (caproic) acid	979	977	[33]	98%	-	-	-	-	0.02	0.06
36	Octanoic (caprylic) acid	1181	1180	[33]	96%	-	-	-	0.03	0.11	0.16
44	Nonanoic (pelargonic) acid	1290	1290	[33]	97%	-	-	-	-	-	0.34
49	Methyl decanoate	1330	1328	[33]	95%	-	-	-	-	0.13	-
51	Decanoic (capric) acid	1376	1377	[33]	98%	1.41	-	0.34	0.34	2.82	0.91
53	Ethyl decanoate	1399	1397	[33]	96%	-	-	-	-	0.42	-
63	Dodecanoic (lauric) acid	1570	1570	[33]	96%	3.78	-	1.85	1.18	2.56	3.90
64	Ethyl dodecanoate	1599	1597	[33]	97%	-	-	-	-	0.65	-
65	Tetradecanoic (myristic) acid	1780	1780	[33]	98%	31.92	7.27	97.01	37.12	75.11	4.20
68	Hexadecanoic (palmitic) acid	1966	1963	[33]	99%	-	-	-	-	-	35.48
71	(*Z*,*Z*)-Octadeca-9,12-dienoic (linoleic) acid	2150	2147	[33]	98%	-	-	-	-	-	40.69
	Total identified (%)					37.11	7.27	99.20	38.67	81.82	85.74
Aliphatic and Aromatic Ketones											
11	6-Methylhept-5-en-2-one	992	991	[33]	99%	0.61	1.51	0.02	0.05	0.11	0.05
23	Acetophenone	1074	1072	[33]	97%	-	-	-	-	-	0.01
30	6-Methyl-3,5-heptadien-2-one	1110	1107	[33]	96%	-	0.26	-	-	-	-
45	Undecan-2-one	1297	1296	[33]	97%	-	-	-	0.10	-	-
58	Acetovanillone	1491	1491	[33]	98%	-	-	-	-	0.12	-
66	Hexahydrofarnesyl acetone	1851	1850	[33]	97%	-	-	-	-	-	0.63
	Total identified (%)					0.61	1.77	0.02	0.15	0.23	0.69
Furan											
12	2-Pentylfuran	997	998	[33]	97%	-	-	-	0.03	-	0.05
Oxygenated Monoterpenes											
17	1,8-Cineole	1041	1037	[33]	98%	0.64	-	-	0.03	0.12	-
25	Dihydromyrcenol	1078	1075	[33]	96%	-	-	-	-	0.02	0.03
26	*trans*-Linalool oxide	1080	1081	[33]	95%	-	0.11	-	-	-	-
28	Linalool	1104	1102	[33]	98%	-	-	-	0.02	0.10	0.04
29	Camphor	1152	1149	[33]	99%	-	-	-	-	0.11	0.04
34	Borneol	1173	1172	[33]	98%	-	-	-	0.02	-	-
37	Terpinen-4-ol	1184	1184	[33]	97%	7.29	-	-	0.02	0.07	0.07
38	α-Terpineol	1196	1195	[33]	98%	3.38	-	-	0.07	0.19	0.06
39	Myrtenol	1199	1198	[33]	97%	9.60	-	-	0.04	0.14	-
42	(*E*)-Citral	1276	1278	[33]	95%	1.49	0.15	-	0.06	0.20	0.03
43	Perilla aldehyde	1279	1279	[33]	98%	-	-	-	-	0.16	0.07
50	Eugenol	1363	1365	[33]	98%	-	-	-	-	-	0.02
56	(*E*)-Geranylacetone	1458	1458	[33]	98%	-	0.21	-	0.02	0.09	0.15
	Total identified (%)					22.40	0.47	-	0.28	1.20	0.51
Norisoprenoids											
40	Safranal	1204	1205	[33]	96%	-	-	-	-	0.20	-
60	*trans*-α-Irone	1520	1504	[33]	97%	-	0.26	-	-	-	-
61	*cis*-α-Irone	1544	1546	[33]	98%	-	24.70	-	-	0.03	0.04
62	*cis*-γ-Irone *	1551	-	[24]	95%	8.43	4.48	-	-	0.08	0.03
	Total identified (%)					8.43	29.44	-	-	0.31	0.07
Sesquiterpene Hydrocarbons											
52	α-Copaene	1381	1376	[33]	97%	-	-	0.02	-	-	-
55	*trans*-Caryophyllene	1424	1423	[33]	99%	-	0.12	-	-	-	-
	Total identified (%)					-	0.12	0.02	-	-	-
Aromatic Esters											
67	Diisobutyl phthalate	1873	1873	[33]	98%	4.13	-	-	2.58	-	1.15
69	Dibutyl phthalate	1967	1967	[33]	97%	1.51	0.39	-	-	-	1.93
	Total identified (%)					5.64	0.39	-	2.58	-	3.08
	Total amount of identified compounds (%)					89.83	90.81	99.50	97.87	89.31	94.12

*—Tentatively identified based on the mass spectrum; RI—calculated (experimental) retention index; RI_L_—retention index from the literature (references); SI—selectivity index (the highest probability of the experimental mass spectrum matching the mass spectrum present in the reference library).

**Table 4:** Sample codes, harvesting locations, collection dates, and geographic coordinates for investigated *Iris* taxa.

Taxon	Sample Code	Location	Collection Date	Latitude/Longitude
*Iris* *pseudopallida*	*I. pseudopallida* B	Bast	28 April 2019	43°19′58.4″ N/ 16°59′2.4″ E
	*I. pseudopallida* D	Dubrovnik	7 April 2019	42°39′36.4″ N/ 18°04′01.7″ E
	*I. pseudopallida* T	Topići	28 April 2019	43°21′46.0″ N/ 16°57′28.4″ E
*Iris* *illyrica*	*I. illyrica* V	Vir	23 April 2019	44°18′27.3″ N/ 15°1′52.5″ E
	*I. illyrica* Z	Zaton	19 April 2019	43°46′26.5″ N/ 15°50′10.8″ E
*Iris* *adriatica*	*I. adriatica*	Brač	4 April 2019	43°21′30.3″ N/ 16°35′49.6″ E

## Data Availability

Data are contained within the article and Supplementary Materials.

## Supplementary Materials

The following supporting information can be downloaded at https://www.mdpi.com/article/10.3390/molecules29174107/s1, Table S1: Abbreviations, molecular formulas, molecular weights (MWs), retention indices (RIs), and average percentages of major volatile organic compounds (VOCs) of the six investigated *Iris* samples, obtained after three different extraction methods; Figure S1: Total ion chromatograms (TICs) of *I. pseudopallida* B: (a) headspace solid-phase microextraction (HS–SPME) using polydimethylsiloxane/divinylbenzene (PDMS/DVB) fiber, (b) HS–SPME using divinylbenzene/carboxene/polydimethylsiloxane (DVB/CAR/PDMS) fiber, and (c) hydrodistillation (HD).
