# Supplementary material for: Gas Chromatography–Mass Spectrometry Analysis of Volatile Organic Compounds from Three Endemic Iris Taxa: Headspace Solid-Phase Microextraction vs. Hydrodistillation
Source: Molecules. 2024 Aug 29;29(17):4107. doi: 10.3390/molecules29174107 (PMC11397506; doi:10.3390/molecules29174107)
Supplement: Supplementary file 1 [file molecules-29-04107-s001.zip › molecules-3169141-supplementary.pdf]

molecules

MDPI

Table S1. Abbreviations, molecular formulas, molecular weights (MWs), retention indices (RIs) and average percentages of major volatile organic compounds (VOCs) of the six investigated *Iris* samples, obtained after three different extraction methods.

Compound	Abbreviation	Molecular Formula and MW	RI	*I. pseudopallida* B	*I. pseudopallida* D	*I. pseudopallida* T	*I. illyrica* V	*I. illyrica* Z	*I. adriatica*
Acetic acid	ACA	$C_2H_4O_2$<br>60.05	<900	2.88[1]<br>14.29[2]<br>0[3]	0[1]<br>0[2]<br>0[3]	6.29[1]<br>29.76[2]<br>0[3]	6.95[1]<br>29.20[2]<br>0[3]	1.91[1]<br>31.02[2]<br>0[3]	3.74[1]<br>12.00[2]<br>0[3]
Acetoin	ACT	$C_4H_8O_2$<br>88.10	<900	0[1]<br>0[2]<br>0[3]	0[1]<br>0[2]<br>0[3]	2.84[1]<br>2.01[2]<br>0[3]	2.86[1]<br>2.42[2]<br>0[3]	1.39[1]<br>5.27[2]<br>0[3]	1.04[1]<br>2.11[2]<br>0[3]
Acetovanillone	ACV	$C_9H_{10}O_3$<br>116.17	1491	0.11[1]<br>0.41[2]<br>0[3]	0[1]<br>0[2]<br>0[3]	0[1]<br>0[2]<br>0[3]	7.12[1]<br>3.85[2]<br>0[3]	0[1]<br>0[2]<br>0.12[3]	0[1]<br>0[2]<br>0[3]
Benzyl alcohol	BEA	C_7H_8O<br>108.14	1042	2.03[1]<br>1.52[2]<br>0[3]	0[1]<br>0[2]<br>0.09[3]	5.46[1]<br>5.08[2]<br>0[3]	3.40[1]<br>1.58[2]<br>0[3]	2.46[1]<br>1.23[2]<br>0[3]	2.40[1]<br>1.62[2]<br>0.04[3]
Butan-2,3-diol	BUD	$C_4H_{10}O_2$<br>90.12	<900	1.78[1]<br>2.41[2]<br>0[3]	0[1]<br>0[2]<br>0[3]	6.38[1]<br>7.05[2]<br>0[3]	5.29[1]<br>7.83[2]<br>0[3]	10.27[1]<br>11.11[2]<br>0[3]	2.18[1]<br>2.43[2]<br>0[3]
Camphor	CAM	$C_{10}H_{16}O$<br>152.23	1152	0[1]<br>0[2]<br>0[3]	0[1]<br>0[2]<br>0[3]	0[1]<br>0[2]<br>0[3]	0[1]<br>0[2]<br>0[3]	0[1]<br>0[2]<br>0.11[3]	3.25[1]<br>2.98[2]<br>0.04[3]
trans-Caryophyllene	TCA	$C_{15}H_{24}$<br>204.36	1424	3.40[1]<br>2.70[2]<br>0[3]	7.24[1]<br>5.50[2]<br>0.12[3]	1.51[1]<br>0.61[2]<br>0[3]	1.97[1]<br>0.82[2]<br>0[3]	2.67[1]<br>1.37[2]<br>0[3]	4.51[1]<br>2.79[2]<br>0[3]

Table S1. Cont.

1,8-Cineole	CIN	$C_{10}H_{18}O$<br>154.25	1041	0.68[1]<br>0.12[2]<br>0.64[3]	0[1]<br>0[2]<br>0[3]	2.65[1]<br>0.56[2]<br>0[3]	0.34[1]<br>0.57[2]<br>0.03[3]	1.16[1]<br>0.75[2]<br>0.12[3]	3.82[1]<br>2.38[2]<br>0[3]
α-Copaene	ACO	$C_{15}H_{24}$<br>204.36	1381	5.14[1]<br>5.06[2]<br>0[3]	1.21[1]<br>0.98[2]<br>0[3]	2.02[1]<br>1.32[2]<br>0.02[3]	3.62[1]<br>2.08[2]<br>0[3]	2.61[1]<br>1.62[2]<br>0[3]	0[1]<br>0[2]<br>0[3]
Decanal	DEA	$C_{10}H_{20}O$<br>156.00	1210	0.62[1]<br>0.50[2]<br>0[3]	0[1]<br>0[2]<br>0.09[3]	2.35[1]<br>1.46[2]<br>0[3]	0[1]<br>0.17[2]<br>0[3]	0.74[1]<br>0[2]<br>0.05[3]	0.49[1]<br>0.36[2]<br>0.04[3]
Decanoic acid	DCA	$C_{10}H_{20}O_2$<br>172.26	1376	0[1]<br>0[2]<br>1.41[3]	0[1]<br>0[2]<br>0[3]	0[1]<br>0[2]<br>0.34[3]	0[1]<br>0[2]<br>0.34[3]	0[1]<br>0[2]<br>2.82[3]	0[1]<br>0[2]<br>0.91[3]
Dihydromyrcenol	DHM	$C_{10}H_{20}O$<br>156.27	1078	0.95[1]<br>1.06[2]<br>0[3]	0[1]<br>0[2]<br>0[3]	3.03[1]<br>1.42[2]<br>0[3]	1.60[1]<br>0[2]<br>0[3]	3.46[1]<br>2.09[2]<br>0.02[3]	2.45[1]<br>2.07[2]<br>0.03[3]
Diisobutyl phthalate	DIBP	$C_{16}H_{22}O_4$<br>278.35	1873	0[1]<br>0[2]<br>4.13[3]	0[1]<br>0[2]<br>0[3]	0[1]<br>0[2]<br>0[3]	0[1]<br>0[2]<br>2.58[3]	0[1]<br>0[2]<br>0[3]	0[1]<br>0[2]<br>1.15[3]
Docosane	DOC	$C_{22}H_{46}$<br>310.60	2200	0[1]<br>0[2]<br>0[3]	0[1]<br>0[2]<br>45.79[3]	0[1]<br>0[2]<br>0[3]	0[1]<br>0[2]<br>55.45[3]	0[1]<br>0[2]<br>1.04[3]	0[1]<br>0[2]<br>0.21[3]
Dodecanoic acid	DDA	$C_{12}H_{24}O_2$<br>200.32	1570	0[1]<br>0[2]<br>3.78[3]	0[1]<br>0[2]<br>0[3]	0[1]<br>0[2]<br>1.85[3]	0[1]<br>0[2]<br>1.18[3]	0[1]<br>0[2]<br>2.56[3]	0[1]<br>0[2]<br>3.90[3]
Ethanol	ETH	C_2H_6O<br>46.07	<900	2.21[1]<br>4.75[2]<br>0[3]	1.39[1]<br>3.22[2]<br>0[3]	6.86[1]<br>4.70[2]<br>0[3]	4.79[1]<br>5.23[2]<br>0[3]	3.65[1]<br>4.34[2]<br>0[3]	2.66[1]<br>7.01[2]<br>0[3]

Table S1. Cont.

(Furan-2-yl)methanol	FUM	$C_5H_6O_2$<br>98.10	<900	0[1]<br>0[2]<br>0[3]	0[1]<br>0[2]<br>0[3]	0[1]<br>0[2]<br>0[3]	0[1]<br>0[2]<br>0[3]	0[1]<br>0[2]<br>0[3]	2.75[1]<br>3.08[2]<br>0[3]
Furfural	FUR	$C_5H_4O_2$<br>96.08	<900	0[1]<br>0[2]<br>1.07[3]	0[1]<br>0[2]<br>0.05[3]	0[1]<br>0[2]<br>0[3]	0.31[1]<br>1.03[2]<br>0.01[3]	0.34[1]<br>0.11[2]<br>0.06[3]	1.80[1]<br>7.91[2]<br>0.02[3]
Hexadecanoic acid	HDA	$C_{16}H_{32}O_2$<br>256.43	1966	0[1]<br>0[2]<br>0[3]	0[1]<br>0[2]<br>0[3]	0[1]<br>0[2]<br>0[3]	0[1]<br>0[2]<br>0[3]	0[1]<br>0[2]<br>0[3]	0[1]<br>0[2]<br>35.48[3]
Hexanal	HEA	$C_6H_{12}O$<br>100.16	<900	0.81[1]<br>1.46[2]<br>0[3]	0[1]<br>0[2]<br>0[3]	0.71[1]<br>1.49[2]<br>0[3]	2.57[1]<br>4.67[2]<br>0[3]	1.14[1]<br>1.30[2]<br>0[3]	0.96[1]<br>1.04[2]<br>0[3]
cis-α-Irone	AIR	$C_{14}H_{22}O$<br>206.32	1544	0[1]<br>0[2]<br>0[3]	45.76[1]<br>43.74[2]<br>24.70[3]	0[1]<br>0[2]<br>0[3]	0[1]<br>0[2]<br>0[3]	0[1]<br>0[2]<br>0.03[3]	0[1]<br>0[2]<br>0.04[3]
cis-γ-Irone	GIR	$C_{14}H_{22}O$<br>206.32	1551	1.87[1]<br>1.99[2]<br>8.43[3]	7.17[1]<br>7.87[2]<br>4.48[3]	0[1]<br>0[2]<br>0[3]	0[1]<br>0[2]<br>0[3]	0[1]<br>0[2]<br>0.08[3]	0[1]<br>0[2]<br>0.03[3]
Limonene	LIM	$C_{10}H_{16}$<br>136.24	1037	1.46[1]<br>1.40[2]<br>0[3]	0[1]<br>0[2]<br>0[3]	0.90[1]<br>0.42[2]<br>0[3]	0.88[1]<br>0.60[2]<br>0.01[3]	0.57[1]<br>0.20[2]<br>0.02[3]	4.79[1]<br>3.52[2]<br>0[3]
Linalool	LIN	$C_{10}H_{18}O$<br>154.25	1104	1.83[1]<br>1.31[2]<br>0[3]	0[1]<br>0[2]<br>0[3]	1.91[1]<br>0.79[2]<br>0[3]	0.75[1]<br>0[2]<br>0.02[3]	1.64[1]<br>0.79[2]<br>0.10[3]	2.57[1]<br>2.20[2]<br>0.04[3]
6-Methylhept-5-en-2-one	MEH	$C_8H_{14}O$<br>126.20	992	1.98[1]<br>2.61[2]<br>0.61[3]	3.59[1]<br>9.10[2]<br>1.51[3]	1.61[1]<br>1.30[2]<br>0.02[3]	3.21[1]<br>3.14[2]<br>0.05[3]	9.47[1]<br>5.83[2]<br>0.11[3]	1.33[1]<br>0.81[2]<br>0.05[3]

Table S1. Cont.

Myrtenol	MYR	$C_{10}H_{16}O$<br>152.23	1199	6.33[1]<br>6.04[2]<br>9.60[3]	0[1]<br>0[2]<br>0[3]	0[1]<br>0[2]<br>0[3]	0[1]<br>0[2]<br>0.04[3]	0[1]<br>0[2]<br>0.14[3]	0[1]<br>0[2]<br>0[3]
Neryl formate	NEF	$C_{11}H_{18}O_2$<br>182.26	1262	0[1]<br>0[2]<br>0[3]	0[1]<br>0[2]<br>0[3]	4.02[1]<br>2.52[2]<br>0[3]	0[1]<br>0[2]<br>0[3]	0[1]<br>0[2]<br>0[3]	1.20[1]<br>1.01[2]<br>0[3]
Nonanal	NOA	$C_9H_{18}O$<br>142.24	1109	2.78[1]<br>2.78[2]<br>0[3]	0[1]<br>0[2]<br>0[3]	3.25[1]<br>2.71[2]<br>0[3]	3.55[1]<br>2.07[2]<br>0[3]	3.10[1]<br>1.24[2]<br>0[3]	1.22[1]<br>0.90[2]<br>0[3]
Nonan-2-one	NON	$C_9H_{18}O$<br>142.24	1091	0[1]<br>0[2]<br>0[3]	4.99[1]<br>5.11[2]<br>0[3]	0[1]<br>0[2]<br>0[3]	0[1]<br>0[2]<br>0[3]	0[1]<br>0[2]<br>0[3]	0[1]<br>0[2]<br>0[3]
(*Z*,*Z*)-Octadeca-9,12-dienoic acid	ODA	$C_{18}H_{32}O$<br>280.45	2150	0[1]<br>0[2]<br>0[3]	0[1]<br>0[2]<br>0[3]	0[1]<br>0[2]<br>0[3]	0[1]<br>0[2]<br>0[3]	0[1]<br>0[2]<br>0[3]	0[1]<br>0[2]<br>40.69[3]
Octanoic acid	OCA	$C_8H_{16}O_2$<br>144.21	1181	0[1]<br>0[2]<br>0[3]	0[1]<br>0[2]<br>0[3]	2.15[1]<br>2.35[2]<br>0[3]	0[1]<br>0[2]<br>0.03[3]	0[1]<br>0[2]<br>0.11[3]	0[1]<br>0[2]<br>0.16[3]
Pentanal	PEA	$C_5H_{10}O$<br>86.13	<900	0[1]<br>0[2]<br>0[3]	3.28[1]<br>0[2]<br>0[3]	0[1]<br>0[2]<br>0[3]	2.48[1]<br>2.65[2]<br>0[3]	0[1]<br>0[2]<br>0[3]	0[1]<br>2.31[2]<br>0[3]
2-Pentylfuran	PEF	$C_9H_{14}O$<br>138.21	997	0.53[1]<br>0.80[2]<br>0[3]	0.84[1]<br>2.37[2]<br>0[3]	0[1]<br>0[2]<br>0[3]	0.58[1]<br>0.96[2]<br>0[3]	1.36[1]<br>0.69[2]<br>0[3]	0[1]<br>0[2]<br>0[3]
Perilla aldehyde	PER	$C_{10}H_{14}O$<br>150.22	1279	20.55[1]<br>15.63[2]<br>0[3]	0[1]<br>0[2]<br>0[3]	17.76[1]<br>8.09[2]<br>0[3]	19.72[1]<br>6.47[2]<br>0[3]	30.00[1]<br>17.59[2]<br>0.16[3]	26.83[1]<br>20.08[2]<br>0.07[3]

Table S1. Cont.

2-Phenylethanol	PHE	$C_8H_{10}O$<br>122.16	1120	4.67[1]<br>4.31[2]<br>2.20[3]	0[1]<br>0[2]<br>0[3]	1.76[1]<br>1.38[2]<br>0[3]	1.70[1]<br>0.77[2]<br>0.02[3]	4.76[1]<br>3.36[2]<br>0.81[3]	0.90[1]<br>0.83[2]<br>0.07[3]
α-Pinene	API	$C_{10}H_{16}$<br>136.24	945	4.48[1]<br>2.46[2]<br>5.98[3]	0[1]<br>0[2]<br>0[3]	0.20[1]<br>0[2]<br>0.01[3]	2.40[1]<br>1.55[2]<br>0.16[3]	1.11[1]<br>0.37[2]<br>0.08[3]	0[1]<br>0[2]<br>0[3]
Terpinen-4-ol	TRN	$C_{10}H_{18}O$<br>154.25	1184	0.33[1]<br>0.41[2]<br>7.29[3]	0[1]<br>0.39[2]<br>0[3]	0.50[1]<br>0[2]<br>0[3]	0[1]<br>0[2]<br>0.02[3]	0[1]<br>0[2]<br>0.07[3]	0.51[1]<br>0.56[2]<br>0.07[3]
α-Terpineol	TER	$C_{10}H_{18}O$<br>154.25	1196	3.19[1]<br>3.01[2]<br>3.38[3]	0[1]<br>0[2]<br>0[3]	1.53[1]<br>1.14[2]<br>0[3]	0.66[1]<br>0.25[2]<br>0.07[3]	0[1]<br>0[2]<br>0.19[3]	0.76[1]<br>0.64[2]<br>0.06[3]
Tetradecanoic acid	TDA	$C_{14}H_{28}O_2$<br>228.37	1780	0[1]<br>0[2]<br>31.92[3]	0[1]<br>0[2]<br>7.27[3]	0[1]<br>0[2]<br>97.01[3]	0[1]<br>0[2]<br>37.12[3]	0[1]<br>0[2]<br>75.11[3]	0[1]<br>0[2]<br>4.20[3]
Undecan-2-one	UND	$C_{11}H_{22}O$<br>170.30	1297	0.43[1]<br>0.21[2]<br>0[3]	2.01[1]<br>1.61[2]<br>0[3]	1.28[1]<br>0[2]<br>0[3]	1.60[1]<br>0.63[2]<br>0.10[3]	0.43[1]<br>0[2]<br>0[3]	0.13[1]<br>0.12[2]<br>0[3]

[1] VOCs obtained by polydimethylsiloxane/divinylbenzene (PDMS/DVB) fiber; [2] VOCs obtained by divinylbenzene/carboxene/polydimethylsiloxane (DVB/CAR/PDMS) fiber; [3] VOCs obtained by hydrodistillation (HD).

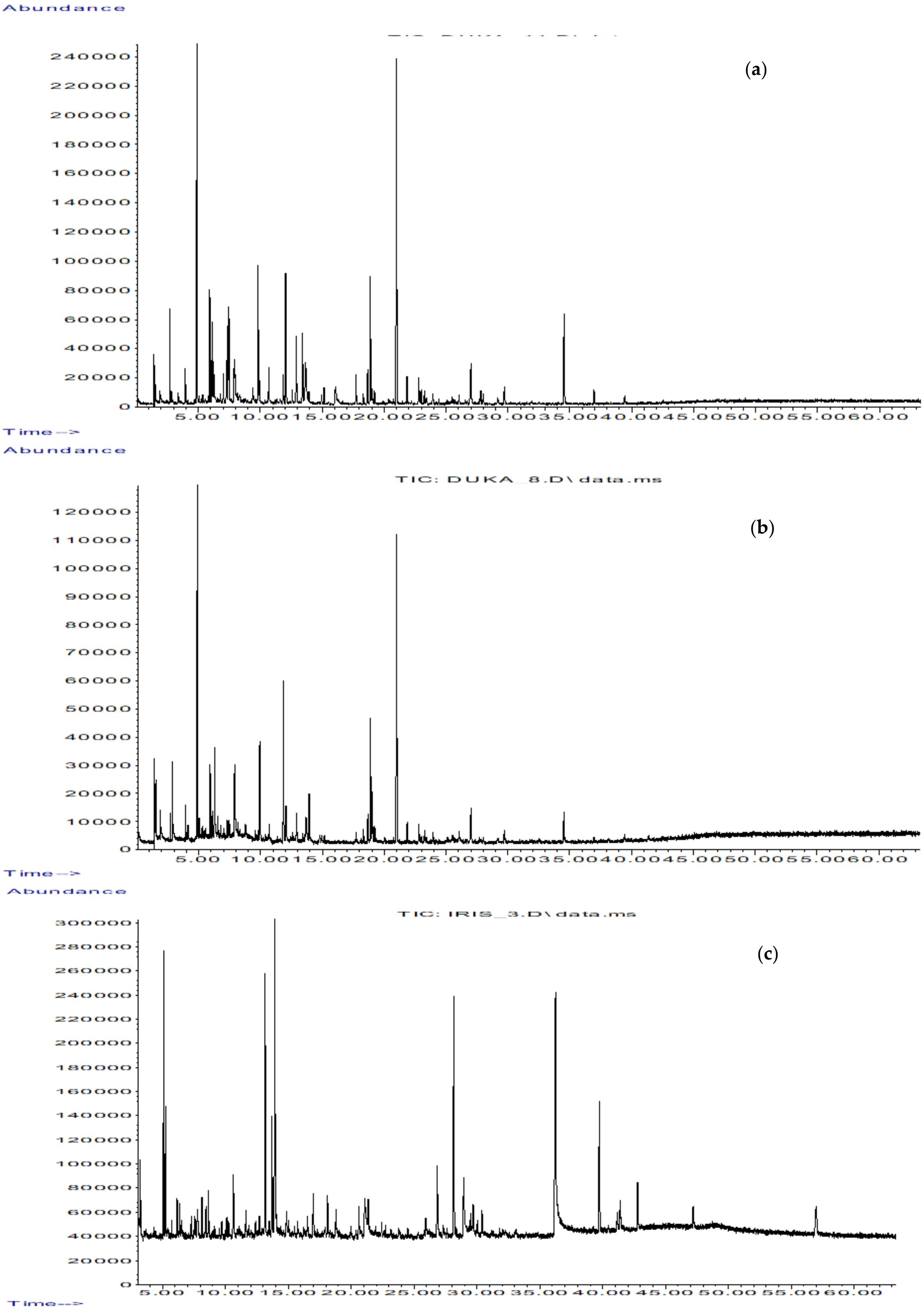

Figure S1. Total ion chromatograms (TICs) of *I. pseudopallida* B: (**a**) headspace solid-phase microextraction (HS–SPME) using polydimethylsiloxane/divinylbenzene (PDMS/DVB) fiber, (**b**) HS–SPME using divinylbenzene/carboxene/polydimethylsiloxane (DVB/CAR/PDMS) fiber, and (**c**) hydrodistillation (HD).
